# The proliferation of atypical hepatocytes and CDT1 expression in noncancerous tissue are associated with the postoperative recurrence of hepatocellular carcinoma

**DOI:** 10.1038/s41598-022-25201-6

**Published:** 2022-11-28

**Authors:** Mitsuhiko Moriyama, Tatsuo Kanda, Yutaka Midorikawa, Hiroshi Matsumura, Ryota Masuzaki, Hitomi Nakamura, Masahiro Ogawa, Shunichi Matsuoka, Toshikatu Shibata, Motomi Yamazaki, Kazumichi Kuroda, Hisashi Nakayama, Tokio Higaki, Kazunori Kanemaru, Toshio Miki, Masahiko Sugitani, Tadatoshi Takayama

**Affiliations:** 1 Division of Gastroenterology and Hepatology, Department of Medicine, Nihon University School of Medicine, 30-1 Oyaguchi-Kamimachi, Itabashi-Ku, Tokyo, 173-8610 Japan; 2 Department of Digestive Surgery, Nihon University School of Medicine, 30-1 Oyaguchi-Kamimachi, Itabashi-Ku, Tokyo, 173-8610 Japan; 3 Department of Physiology, Division of Biomedical Sciences, Nihon University School of Medicine, 30-1 Oyaguchi-Kamimachi, Itabashi-Ku, Tokyo, 173-8610 Japan; 4 Department of Pathology, Nihon University School of Medicine, 30-1 Oyaguchi-Kamimachi, Itabashi-Ku, Tokyo, 173-8610 Japan

**Keywords:** Cancer, Biomarkers, Oncology, Risk factors

## Abstract

Recently, we reported that extent of proliferation of atypical hepatocytes (atypical hepatocytes) was most important histological risk factor for development of hepatocellular carcinoma (HCC) from chronic hepatitis C or liver cirrhosis. Here, we aimed to clarify whether the atypical hepatocytes in noncancerous sections is also involved in postoperative recurrence. Furthermore, we investigated significant genes involved in the atypical hepatocytes. Association between the extent of atypical hepatocytes in noncancerous tissue and postoperative recurrence was validated in 356 patients with HCC. Next, we identified putative signature genes involved in extent of atypical hepatocytes. First, atypical hepatocytes or hepatocytes other than the atypical hepatocyte in noncancerous sections of 4 HCC patients were selectively collected by laser capture microdissection (LCM). Second, the gene expression profiles of the selected hepatocyte populations were compared using Ion AmpliSeq Transcriptome Human Gene Expression Kit (Thermo Fisher SCIENTIFIC, Waltham, MA, USA) analysis. Finally, we validated the mRNA expression of the extracted genes in noncancerous frozen liver tissue from 62 patients with HCC by RT-qPCR to identify the signature genes involved in both the extent of atypical hepatocytes and postoperative recurrence. Furthermore, the extent of atypical hepatocytes and CDT1 expression in noncancerous sections from 8 patients with HCC were also validated by selectively collecting samples using LCM. The extent of atypical hepatocytes was associated with postoperative recurrence. Of the genes that showed significant differences in expression levels between two populations, the expression of the chromatin licensing and DNA replication factor 1 (*CDT1*) gene was most strongly associated with the extent of atypical hepatocytes and was also associated with postoperative recurrence. Furthermore, CDT1-positive cells that exhibited stronger expression resembled those morphologically considered to be atypical hepatocytes. CDT1 and Ki-67 were colocalized in the nuclei of both hepatocytes and cancer cells. The hepatocytes in noncancerous livers were not uniform in each hepatocyte population, suggesting that the accumulation of genetic abnormalities was variable. We found that the strong degree of atypical hepatocytes and high *CDT1* mRNA expression represent a high carcinogenic state of the liver. Thus, we consider the evaluation of degree of these could support the personalized medicine.

## Introduction

The prognosis of patients with hepatocellular carcinoma (HCC) remains poor, with a 10-year survival rate of ~ 10%^1^, in part due to frequent intrahepatic recurrence and baseline liver dysfunction. Therefore, early prediction and prevention of HCC as well as its recurrence are crucial. There have been various studies on risk factors for HCC development and recurrence^2–4^. However, the driver genes underlying the occurrence of HCC have not yet been identified by whole-genome sequencing or genome-wide association studies. To date, no clinically useful genes for the early prediction and prevention of HCC development have been identified from studies using cancer tissues. On the other hands, It has been hypothesized, although not confirmed, that large- and small-cell liver dysplasia is a histological feature of preneoplastic lesions of HCC in hematoxylin and eosin (HE) stained section by histological studies^5,6^. To improve the prognosis of HCC, we consider that it is important to elucidate the mechanisms by studying the carcinogenic process. From this perspective, it is important to identify histological findings associated with HCC development in noncancerous tissues rather than cancerous tissues to improve the prognosis of HCC, that is, to determine the hepatocyte features that would be associated with preneoplastic lesions.

Regarding histological factors in HE section thought to be associated with carcinogenesis in noncancerous liver section, the degree of irregular regeneration (IR) of hepatocytes has been reported by Uchida et al.^7–11^. The theory of the IR of hepatocytes was proposed by Peters^11^ and developed by Uchida^8–10^. The IR of hepatocytes in lobules is a general term encompassing “anisocytosis of hepatocytes,” “map-like distribution,” “nodular arrangement of parenchyma,” “oncocytic change of hepatocytes,” “proliferation of atypical hepatocytes (atypical hepatocytes),” and “bulging of hepatocytes” (Supplementary Fig. S1). The IR of hepatocytes is thus defined as including six different hepatocyte morphologies, each of which forms a cluster. However, the association between the grade of these six different hepatocyte groups and the occurrence of HCC has not yet been clarified. We defined the grade of IR comprehensively for six different hepatocyte groups and showed that the grades of IR of hepatocytes are a predictive factor for HCC development^7,12^.

**Figure 1:**
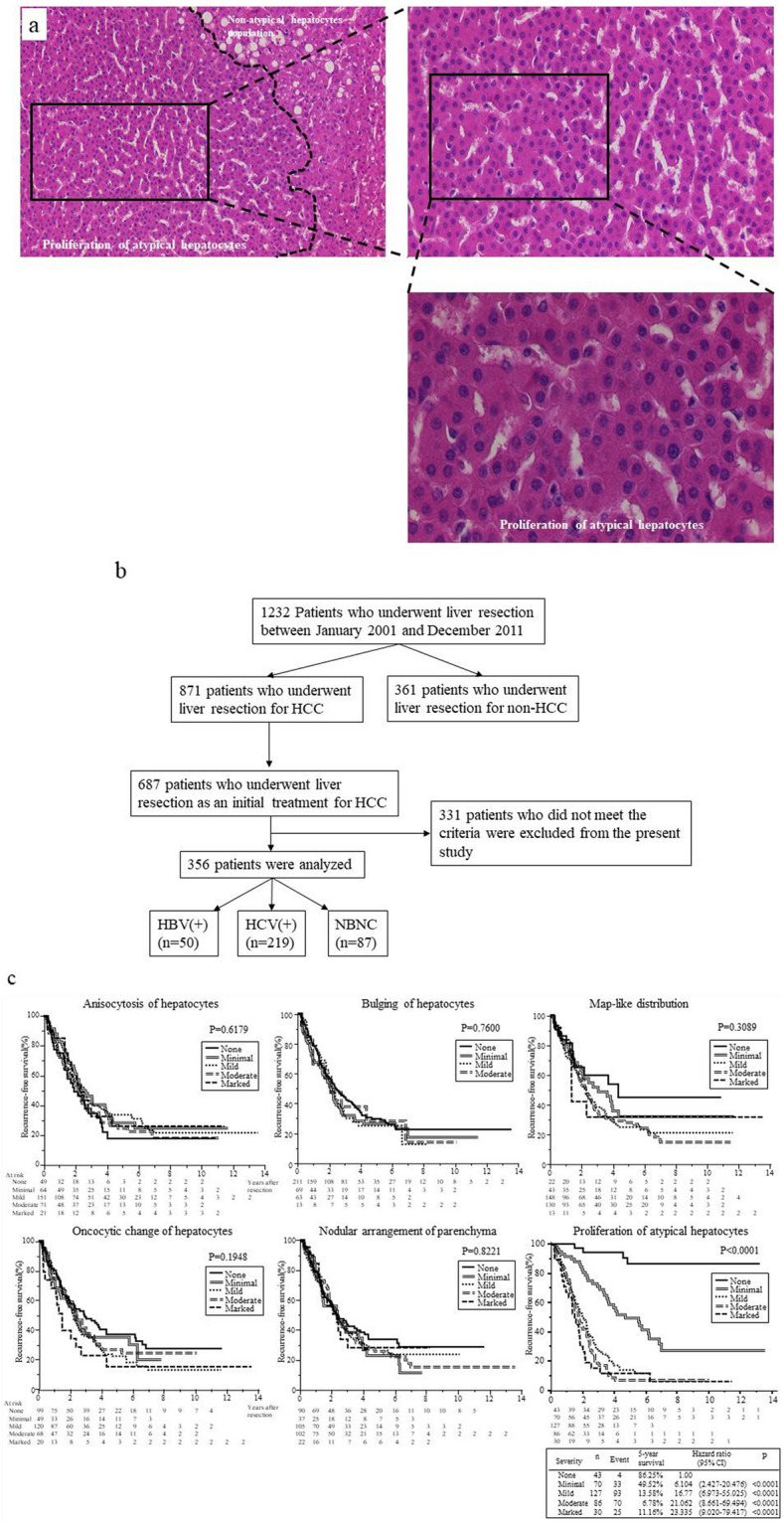
Images of proliferation of atypical hepatocytes. (**a**) Low magnification image of atypical hepatocyte cluster (upper left image; hematoxylin and eosin (HE) staining, × 40). Moderate magnification image of the insert in the upper left panel. The area surrounded by the dashed line encloses an atypical hepatocyte cluster (upper right image; HE, × 200). High magnification image of upper right pane show the atypical hepatocyte cluster (bottom right image; HE, × 400). (**b**) The patients enrolled in Cohort 1 in this study. Based on the inclusion criteria, 331 of 687 patients were excluded. (**c**) Comparison of the cumulative incidence of recurrence-free survival (RFS) rates according to the different grade of irregular regeneration of hepatocytes of the six hepatocyte groups in noncancerous HE-staining sections. Anisocytosis of hepatocytes (p = 0.6179); bulging of hepatocytes (p = 0.75); map-like distribution (p = 0.3089); oncocytic change of hepatocytes (p = 0.1948); nodular arrangement of parenchyma (p = 0.8221); proliferation of atypical hepatocytes (p < 0.0001). Data were analyzed using the Kaplan–Meier method, and differences among groups were analyzed using the logrank test. HBV (+); hepatitis B virus surface antigen-positive, HCV; hepatitis C virus antibody-positive, NBNC, patients without HBsAg or anti-HCV antibody, RT-qPCR, real-time reverse transcription-quantitative PCR.

Recently, we defined the grade according to the IR of each group of hepatocytes and examined the correlations between the grade of IR of hepatocytes and occurrence of HCC in chronic hepatitis C or cirrhosis^13^. This analysis showed that the extent of atypical hepatocytes is a strong histological risk factor for HCC development. The images of atypical hepatocytes represented in Fig. 1a. The following morphological characteristics of atypical hepatocytes in the lobule with HE stained section are noted based on light microscopy: Atypical hepatocytes are usually detected at the light microscopic level as close aggregates of several or more atypical hepatocytes. The characteristics of individual atypical hepatocytes make it possible to distinguish them from other hepatocytes. Furthermore, hepatocytes other than atypical hepatocytes are usually absent or present in limited numbers in a cluster of atypical hepatocytes. Therefore, it is not as difficult to detect close aggregation of atypical hepatocytes in a noncancerous liver tissue section. Furthermore, the morphology of atypical hepatocytes is generally characterized by smaller cells with a higher nucleo-plasmic (N/C) ratio, nuclei that show mild size disparity and cytoplasm with enhanced eosinophilia, a clear distinction between atypical and normal hepatocytes, and no presence of fat droplets. The cluster of atypical hepatocytes is irregularly distributed within the lobule and is not restricted to the periportal area or the pericentral area. There is not only a single population of atypical hepatocytes within the lobule. In contrast, several individual populations of atypical hepatocytes can be present, whereas some lobules are completely devoid of atypical hepatocytes. Lymphocyte infiltration into areas of atypical hepatocyte populations is usually minimal.　There was no difference in the morphological appearance of atypical hepatocytes among Hepatitis B virus (HBV) surface antigen (HBsAg)-positive, hepatitis C virus (HCV) antibody-positive, and HBV- and HCV-negative (non-B non-C; NBNC) patients.

Therefore, we hypothesized that the grade of atypical hepatocytes was the most important histological risk factor in the postoperative recurrence of HCC, as well as in the development of HCC from CHC. Based on this hypothesis, we further hypothesized that the gene expression profile differs between atypical hepatocytes and hepatocytes other than atypical hepatocytes (nonatypical hepatocytes) in the parenchyma and that these differences in gene expression levels could be associated with hepatocarcinogenesis. Thus, the aim of this study was to first determine whether the grade of atypical hepatocytes in the noncancerous liver tissue of surgically resected hepatocellular carcinoma patients is also associated with postoperative recurrence or recurrence time. Second, we investigated whether the gene expression profile of the atypical hepatocytes differs from that of the nonatypical hepatocytes by laser capture microdissection (LCM) and subjected it to transcriptome sequencing (RNA sequencing [RNA-seq]) analysis. The final step was to identify the signature genes associated with the atypical hepatocytes.

## Materials and methods

### Study design

From January, 2001 to October, 2012, a total of 1232 patients, including 871 patients with HCC, underwent liver resection at the Department of Digestive Surgery, Nihon University School of Medicine. We used liver tissues from 678 (77.8%) of the 871 patients who underwent treatment-naïve curative liver resection for HCC and for whom HE-staining microscopic sections of the liver tissue were available. The clinical stage of HCC was determined according to the Japanese General Rules for Primary Liver Cancer^14^. The liver status of the patients was evaluated according to a previously reported classification^12,13^. After the exclusion criteria, 362 out of 678 patients were excluded. Finally, 356 patients were analyzed as cohort 1 to identify whether the grade of atypical hepatocytes was involved in postoperative recurrence and recurrence time (Fig. 1b). The characteristics of the patients are shown in Supplementary Table S1. In addition, signature genes associated with atypical hepatocytes were investigated by Ion AmpliSeq Transcriptome Human Gene Expression Kit (Thermo Fisher SCIENTIFIC, Waltham, MA, USA) using formalin-fixed paraffin-embedded (FFPE) sections of noncancerous livers from 4 HCV (+) patients with HCC who received curative resection between January 1 and December, 2014 as Cohort 2.

Next, to identify signature genes, we examined noncancerous snap-frozen liver tissues or formalin-fixed paraffin-embedded FFPE sections from 62 patients with HCC who underwent treatment-naïve curative resection from February, 2011, to October, 2012, as Cohort 3. Snap-frozen liver tissues were collected from each of these 62 patients and used for real-time reverse transcription-quantitative PCR (RT-qPCR) analysis to identify signature genes involved in the postoperative recurrence of HCC. The clinical characteristics of the 62 patients are summarized in Supplementary Table S2.

We further examined the relationship between the localization of atypical hepatocytes and the signature gene-positive cells in FFPE sections with immunohistochemistry (IHC). Furthermore, we observed whether the signature gene and Ki-67 were colocalized using confocal laser scanning microscopy (CLSM) with noncancerous and cancerous sections in 5 patients with HCV (+) HCC who underwent liver resection from January to March, 2021 as Cohort 4.

Finally, we investigated the relationship between various hepatocyte populations in lobules and signature gene mRNA expression in FFPE sections using LCM. Seven HCV (+) patients who underwent liver resection in 2017 were included as Cohort 5. We evaluated the expression of the extracted signature gene in atypical hepatocytes and hepatocytes other than atypical hepatocytes (nonatypical hepatocytes) and cancer cells using RT-qPCR from the same FFPE sections containing the tumor nodule.

### Exclusion criteria

We used the following exclusion criteria in this study: (1) Patients with clinical stage IVA or IVB and/or macroscopic curability C HCC at surgery. (2) Patients with positive results for any one of the following factors: (a) intrahepatic metastasis (im), (b) bile duct invasion (b), (c) surgical margins (sm), (d) portal vein invasion (vp), (e) hepatic vein invasion (vv), (f) invasion of the serosa (s), and (g) Sm less than 3 mm. (3) Patients receiving adjuvant chemotherapy after surgery, including interferon therapy. (4) Patients younger than 18 years. (5) Patients who underwent a second or more liver resection. These criteria were based on the checklist on handling rules for histopathological findings, Liver Cancer Study Group of Japan (The 6^th^ Edition, Revised Version)^14^.

### Definite diagnosis of postoperative recurrence of HCC

A definite diagnosis of postoperative recurrence of HCC was obtained by abdominal angiography or histological diagnosis of HE sections in tumor tissue, carried out when an HCC nodule was suspected by contrast-enhanced abdominal ultrasonography and/or enhanced computed tomography (CT). All patients were closely monitored for HCC recurrence at each postsurgical outpatient visit (once every 3–6 months after discharge) until March 31, 2021^2^. Patients who dropped out before the completion of the study, owing to either transfer or discontinuation of visits, were also included in the analysis.

Serum HBs antigen (Ag) was quantified using enzyme-linked immune sorbent assay (ELISA; Dinabot Co., Ltd., Tokyo, Japan), and HCV antibodies were quantified using second- or third-generation ELISAs (Abbott Japan LLC, Tokyo, Japan). Habitual alcohol drinkers who consumed over 50 g of ethanol daily were included in the NBNC group. The etiologies were referred to as HBV, HCV, and NBNC, depending on the cause of HCC. The time to postoperative recurrence was defined as the time between liver resection and subsequent diagnosis of any type of relapse, including intrahepatic recurrence.

### Histological evaluation

We evaluated the histological factors in noncancerous HE-staining sections from FFPE tissue of HCC using a scoring system including six different hepatocyte groups with irregular regeneration, inflammatory cell infiltration, fibrosis, and steatosis according to our previous reports without any information on patient characteristics^3,7,12,13^. From each patient with HCC in Cohort 1, two sections of noncancerous HE-staining sections that were not adjacent to a cancerous area were randomly selected and then analyzed by assigning a score to each section (Supplementary Table S3). The noncancerous liver tissues were fixed in 10–20% buffered formalin and embedded in paraffin. The FFPE blocks were sliced into 3–4 µm sections and staining with HE.

The noncancerous and cancerous liver tissues of 62 patients in cohort 3 were divided into two portions immediately after excision, one for snap-frozen and the other for FFPE sections. Further, the histological score was evaluated with FFPE HE- staining sections. The factors of the handling rules for histopathological findings, Liver Cancer Study Group of Japan^14^ were evaluated from the pathological report by the pathologist of our hospital. Histological evaluations were interpreted from March 2011 to July 2014 at a conference centered on M.M. and H.N. without any knowledge of the characteristics of the patients. M.M. was responsible for interpreting the result as in the text, but he is working with co-author H.N. Professor MS finalized the interpretation when the two judges had differing opinions.

M.M. and H.N. have received sufficient education in liver histology from Prof. Toshio Shikata, Prof. Toshikazu Uchida, and Prof. Masahiko Sugitani at the First Department of Pathology, Nihon University^15–20^. They also received detailed education in about the IR of hepatocytes from Professor Toshikazu Uchida, and M.M. has collaborated to these scores and report the parameters of noncancerous liver HE-staining section in patients with HCC^12^. In addition, M.M. has been reported in a number of journals on the relationship between the degree of IR of hepatocytes and the pathogenesis of HCC development^3,13,21–28^.

### Laser-capture microdissection

Using laser captured microdissection (LCM; LMD6500 system, Leica Microsystems, Wetzlar, Germany), we selectively isolated atypical hepatocytes and cells other than atypical hepatocytes (nonatypical hepatocytes) in FFPE sections. LCM resection was performed in 10 of the 72 HCV (+) patients who received curative resection during January and December, 2014, with fibrotic stages of F4 or F3 and moderate or greater grade of atypical hepatocytes with a sufficient area of noncancerous liver FFPE sections. Then, 4 subjects were selected by RNA verification using a Bioanalyzer (Agilent Technologies, Inc., Santa Clara, CA, USA) and used in the following experiments as Cohort 2. Five micrometer-thick sections were obtained from the noncancerous FFPE tissues and placed on a 2.0-µm MembraneSlides membrane (No. 11505158, Leica Microsystems, Tokyo, Japan) with a specific membrane film for laser microdissection. Following deparaffinization with xylene and dehydration with 100% ethanol for 5 min three times, the section was washed with water for 15 min, stained with 0.01% toluidine blue (Sigma-Aldrich Corp., St. Louis, MO, USA) for 2 min without drying, then washed with water again, and air dried for 12 h or more at room temperature (25–28 ℃).

The sections were mounted on a laser-capture microscope (LMD6500 system, Leica Microsystems), and LCM was performed within 72 h of the staining. The micro-dissected sections were collected in tube caps (Thermo Scientific Japan, Yokohama, Japan) with 10 μL of solution no. 1 from the RNeasy FFPE Kit (QIAGEN, Tokyo, Jappan). The dissected cells were collected in an Eppendorf lid. We dissected both the area of aggregated atypical hepatocytes and the area without atypical hepatocytes in the same section (Supplementary Fig. S2a–c). The atypical hepatocytes were collected from an area of the lobule of at least 10 mm^2^. Nonatypical hepatocytes were collected from almost the entire section, excluding atypical hepatocytes, from an area of at least 10 mm^2^. We then excluded the portal and periportal areas and the regions with marked lymphocytic infiltrations and fibrosis because the gene expression of lymphocytes was different from that of hepatocytes.

RNA was extracted from the dissected samples using an RNeasy FFPE Kit (QIAGEN). The quality of the obtained RNA was assessed using an Agilent 2100 Bioanalyzer (Agilent Technologies). The collected RNAs were subjected to Ion AmpliSeq Transcriptome Human Gene Expression Kit (Thermo Fisher SCIENTIFIC, Waltham, MA, USA).

We further investigated for the relationship between various hepatocytes population in lobules and the signature gene mRNA expression in a FFPE section using LCM. The 7 patients with HCV (+) who underwent liver resection in 2017 were used for examination as verification Cohort 5. The 7 patients in liver fibrosis stage were 4 F4 stage (severity of atypical hepatocytes was mild in 3 patients, moderate in 1 patients), 2 F2 stage (severity was minimal in both patients), and 1 F1 stage (severity was minimal). The controls were 7 non-cancerous liver FFPE sections of patients who underwent liver resection for metastatic colorectal cancer in 2017. These control patients were both HCV antibody and HBV core antibody negative, and liver function tests were within the normal range.

### Ion AmpliSeq transcriptome (RNA-seq) analysis

To identify the genes that are characteristically expressed in the atypical hepatocyte population involved in postoperative recurrence, RNA extracted from the selectively collected hepatocytes populations in Cohort 2 were compared using Ion AmpliSeq Transcriptome Human Gene Expression Kit (Thermo Fisher SCIENTIFIC) analysis. Libraries for RNA sequencing were generated using the Ion AmpliSeq Human Gene Expression Kit (Thermo Fisher SCIENTIFIC) according to the manufacturer’s protocol. Briefly, 100 ng of RNA from the FFPE section was reverse-transcribed to cDNA at 80 °C for 10 min. The target genes were amplified using the Ion AmpliSeq Human Gene Expression Panel, which targets ~ 20,802 genes (human reference sequence genes), that is, 18,574 coding genes and 2,228 non-coding genes (based on UCSC hg19). Library concentration and amplicon size were evaluated using a bioanalyzer. The libraries were loaded on the Ion PI Chip (Thermo Fisher SCIENTIFIC) and sequenced using the Ion Torrent Proton sequencing system and the Ion PI Sequencing 200 kit v3 chemistry (Thermo Fisher SCIENTIFIC) according to previous reports^29,30^. The sequencing data were mapped using the Ion Torrent Mapping Alignment Program and normalized using reads per million. Gene expression was analyzed using the GeneSpring GX software (version 2.0; Agilent Technologies).

### RNA extraction and reverse transcription-quantitative PCR (RT-qPCR) analysis

Snap-frozen liver tissues were collected from each of the 62 patients in Cohort 3 and used for RT-qPCR analysis to identify signature genes involved in the degree of atypical hepatocytes and the postoperative recurrence of HCC. In addition, various hepatocyte populations were collected from noncancerous FFPE sections of 7 HCC patients in Cohort 5 using LCM, and signature gene mRNA expression in each cell cluster was examined by RT-qPCR. From each 50 mg of stored frozen liver tissue sample, at least 50 μg of RNA was extracted using TRIzol (Thermo Fisher SCIENTIFIC). cDNA was synthesized using the SuperScript III First-Strand Synthesis System (Thermo Fisher SCIENTIFIC). qPCR was performed using the THUNDERBIRD SYBR qPCR Mix (TOYOBO, Osaka, Japan) on the 7500 Fast Real-Time PCR System (Thermo Fisher SCIENTIFIC) according to the manufacturer’s instructions. The cycling program was 95 °C for 1 min, followed by 40 cycles at 95 °C for 15 s, and 60 °C for 1 min. β-Actin (*ACTB*) was used as an internal control, and data were evaluated via the comparative threshold cycle method. The mRNA expression of each gene was quantified using the formula *ΔCt* = *Ct *_*for each gene test*_*: Ct *_*β-actin*_. RT-qPCR experiments were performed in triplicate. The PCR primers used in the study are listed in Supplementary Table S4.

### Immunohistochemistry

We examined the relationship between the localization of atypical hepatocytes and signature gene (chromatin licensing and DNA replication factor 1; CDT1)-positive cells in FFPE sections using immunohistochemistry from 5 patients in Cohort 4 who underwent liver resection from January 7 to March 20, 2015. In addition, colocalization of Ki-67 and the CDT1 gene was examined by CLSM.

Noncancerous liver tissues were cut into 5-μm-thick sections, formalin fixed, and paraffin embedded. These sections were dewaxed with xylene and dehydrated using a series of ethanol washes. Antigen retrieval was performed with a water bath (BM-41) at 95 ℃ for 20 min in EnVision FLEX Target Retrieval Solution, Low pH 9.0 ([K8005, Dako Omnis] from Agilent Technologies). Endogenous peroxidase activity was blocked using EnVision FLEX blocking reagent (K8023, Dako Omnis, Agilent Technologies) and Block Ace containing 5% normal goat serum (KAC Co., Ltd., Tokyo, Japan) according to the manufacturer’s instructions. A rabbit polyclonal anti-CDT1 antibody (14382-1-AP, Proteintech) and a rabbit monoclonal anti-Ki-67 antibody (27309-1-AP, Proteintech) were incubated at a concentration of 10 μg/mL overnight at 4 °C, and immunodetection was performed using an EnVision FLEX anti-rabbit Ig reagent kit (K8023, Dako, Agilent Technologies). The stained sections were counterstained with hematoxylin solution (FUJIFILM Wako Pure Chemical Industries, Osaka, Japan). Each slide was viewed with a brightfield microscope (OLYMPUS BX43, Tokyo, Japan) or BZ-X710 (Keyence, Osaka, Japan).

### Immunofluorescence study

The FFPE sections in noncancerous liver in Cohort 4, including a part of the cancer nodule, were washed three times with phosphate-buffered saline (PBS; 162-19321, FUJIFILM Wako Pure Chemical Corporation) for 5 min at room temperature. Activation of antigen was performed at 95–100 °C for 20 min using a concentrated antigen activator (pH 9.0 or pH 6.0) (K800004, K8005, 50X Dako, Ajilent Technology) and then left at room temperature for 30 min. Furthermore, the slides were incubated with 5% goat serum in Block Ace Powder (UKB40, Megumilk Snow Brand Co., Tokyo, Japan) at room temperature for 1 h to block nonspecific binding activity. Then, a rabbit polyclonal anti-CDT1 antibody (Proteintech) and a rabbit monoclonal anti-Ki-67 antibody (Proteintech) were incubated at a concentration of 10 μg/mL overnight at 4 °C. Next, the slides were labeled with the antibody (Alexa Fluor™ 488, 594 labeling kit, Thermo Fisher SCIENTIFIC). The slides were reacted with the fluorescently labeled antibody at 4 °C overnight. The slides were washed three times with phosphate-buffered saline (PBS; 162-19321, Fujifilm Wako) for 5 min at room temperature, and finally, the slides were enclosed with Prolong containing 4',6-diamidino-2-phenylindole (DAPI, P36980, ProLong™ Glass Antifade Mountant, Thermo Fisher SCIENTIFIC) diluted in PBS. The primary antibodies used in the IF study were the same as those used for IHC. After immunostaining, we examined the localization of each gene. Furthermore, we performed double immunofluorescence staining of CDT1 and Ki-67 in noncancerous liver FFPE sections. To detect the coexistence of CDT1 and Ki-67, we acquired confocal images with IF staining in paraffin sections with confocal laser scanning microscopy **(**CLSM, TCS SP8, Leica Microsystems, Wetzlar, Germany). IF images were captured with a TCS SP8 confocal microscope using a × 63 HC PL APO oil immersion objective at excitation: 488 nm and emission: 490–545 nm for MitoTracker Green and excitation: 552 nm and emission: 605–655 nm for R-CEPIA3/4mt^31^.

### Cell culture

The human hepatoma cell line Huh7 was purchased from the Japanese Collection of Research Bioresources Cell Bank (Ibaraki, Japan). The cells were maintained with Dulbecco’s modified Eagle’s medium with 10% fetal calf serum (Sigma-Aldrich; Merck KgaA, Darmstadt, Germany) and grown at 37 °C with 5% CO_2_.

### Transfection and RNA extraction

A total of 4 × 10^5^ cells per well were inoculated in 6-well plates 24 h prior to transfection. At 48 h and 72 h post-transfection, total RNA was collected using RNeasy (QIAGEN GmbH), and then, cDNA was synthesized using 2 μg RNA. The siRNAs against CDT1 and control siRNA (Si–C) were purchased from Santa Cruz Biotechnology (Santa Cruz, CA, USA). Transfection of 50 nM siRNAs was performed with GenomONE-Si (Ishihara Sangyo Corporation Osaka, Japan). CDT1 expression was analyzed by Western blotting.

### Western blot analysis

Cell lysates were prepared 48 h post-transfection in 1 × Na Page sample buffer (FUJIFILM Wako Pure Chemical Industries). Cell lysate proteins (5 μg/well) were separated by iBlot (Thermo Fisher SCIENTIFIC). CDT1 expression was analyzed by Western blotting. The membrane was incubated with rabbit polyclonal antibodies against CDT1 (Proteintech) at 4 °C for 16 h. Membranes were then incubated with anti-rabbit IgG HRP-linked secondary antibody (GE Healthcare Life Sciences, Chalfont, UK) at room temperature for 1 h. Then, β-actin expression was incubated with mouse monoclonal antibodies against β-actin (AC-15, Sigma-Aldrich) at 4 °C for 16 h. Membranes were then incubated with anti-mouse/human IgG-HRP (GE) at 4 °C for 16 h. Proteins were visualized using an ECL plus kit (GE Healthcare Life Sciences).

### MTT (3-(4,5-dimethylthiazol-2yl)-2,5-diphenyltetrazolium bromide) assay

Huh7 cell viability was assessed by MTT assays in 96-well plates. After transfection or incubation using SiCDT1 for 24 h, 72 h and 128 h, the cells were incubated with 10 μl of a 5 mg/ml MTT solution at 37 °C for 4 h. The absorbance was read at 450 nm using a spectrophotometer (TECAN Life Sciences, Männedorf, Switzerland).

### Nucleotide sequence accession number

All sequence reads were registered in the Gene Expression Omnibus under accession numbers GSE153742 and GSM4650405-GSM4650412.

### Statistical analysis

Data are shown as the mean ± SD or median (range). Statistical analyses of patient characteristics were conducted using univariate analysis, and for comparisons among multiple groups, Kruskal–Wallis and Steel–Dwass tests were used. Spearman’s rank correlation test was used for correlation analysis among the groups. The cumulative incidence curve was developed with the Kaplan–Meier method, and differences among groups were evaluated using the log-rank test. Risk factors for HCC recurrence were analyzed using a multivariate Cox proportional hazards regression model, with sex, age, etiology, signature gene mRNA expression and histological findings at the time of liver resection as variables. The results with p < 0.01 were considered statistically significant. Statistical analysis was performed using JMP 12 Statistical software (SAS Institute, Inc., Tokyo, Japan).

### Ethics approval and consent to participate

This study protocol complied with the ethical guidelines of the 1975 Declaration of Helsinki and was approved by the Ethics Committee of Nihon University (#131, #241-1, RK-200702-1). Written informed consent was obtained from each patient.

## Results

### The extent of atypical hepatocytes is the most significant histological risk factor for postoperative recurrence

In 356 patients (Cohort 1), the presence or absence of postoperative recurrence and time to postoperative recurrence were independently associated with the extent of atypical hepatocytes (HR = 1.72; 95% CI 1.51–1.96; p < 0.0001) and tumor size (HR = 1.01; 95% CI 1.003–1.03; p = 0.0206; Table 1). The time to postoperative recurrence was significantly related only to the extent of atypical hepatocytes (p < 0.0001; Supplementary Fig. S3). We then investigated the relationship between the time to postoperative recurrence and the extent of six different hepatocyte groups according to the cumulative incidence of RFS. The extent of the six different hepatocyte groups were not correlated with the cumulative incidence of RFS except for the extent of atypical hepatocytes (“atypical hepatocytes,” p < 0.0001. Figure 1c). These data suggested that the extent of atypical hepatocytes was a significant factor for both the presence or absence of recurrence and the time to recurrence of HCC. The cumulative 5-year RFS of HBV (+), HCV (+), and NBNC stratified by extent of atypical hepatocytes are shown in Supplementary Fig. S4. Patients with a higher extent of atypical hepatocytes, regardless of etiology, also had higher HCC recurrence rates. Regardless of the etiology of HCC, the cumulative 5-year RFS were significantly lower in patients with a greater extent of atypical hepatocytes. Thus, the factors contributing to recurrence and time to postoperative recurrence were analyzed using two methods; only the extent of atypical hepatocytes was found to be a significant factor.

**Table 1:** Factors affecting postoperative-recurrence in patients with HCC in Cohort 1 by Cox multivariate proportional hazards regression model. (n = 356).

Parameter	HR	95% CI		P-value
Age	1.0044	0.988	1.021	0.5997
Sex (female)	1.1588	0.820	1.621	0.3988
**Etiology (HCV)**				
HBV	1.249	0.773	2.067	0.3675
NBNC	1.415	0.887	2.289	0.1458
Anisocytosis of hepatocytes	0.8942	0.780	1.026	0.1105
Bulging of hepatocytes	1.0308	0.868	1.217	0.7254
Map-like distribution	1.0094	0.827	1.235	0.9269
Oncocytic change of hepatocytes	1.0404	0.898	1.208	0.5995
Nodular arrangement of parenchyma	1.0336	0.910	1.177	0.6137
Proliferation of atypical hepatocytes	1.7245	1.518	1.962	< 0.0001
Periportal	1.0134	0.764	1.337	0.9256
Parenchymal	1.2054	0.880	1.653	0.2444
Portal	1.0257	0.736	1.420	0.8797
Fibrosis stage	1.0369	0.903	1.194	0.6099
Lymphoid aggregation in portal tract	0.9281	0.780	1.114	0.4167
Bile duct damage	0.9775	0.801	1.169	0.8115
Portal sclerotic change	1.0920	0.816	1.442	0.5472
Pericellular fibrosis	0.9726	0.811	1.165	0.7632
Steatosis	0.9386	0.89	1.090	0.4055
Size	1.0180	1.003	1.032	0.0206

Hazard ratio (HR) was calculated using the Cox multivariate proportional hazards regression model. Peri-venular fibrosis in the subjects of F3 and F4 stages, was excluded from the factor because there were many undetectable in hematoxylin and eosin section.

*HCV, patients with hepatitis c virus antibody-positive; HBV, patients with hepatitis B virus surface antigen-positive; NBNC, patients with both negative.*

### Identification of genes associated with atypical hepatocytes

**Figure 2:**
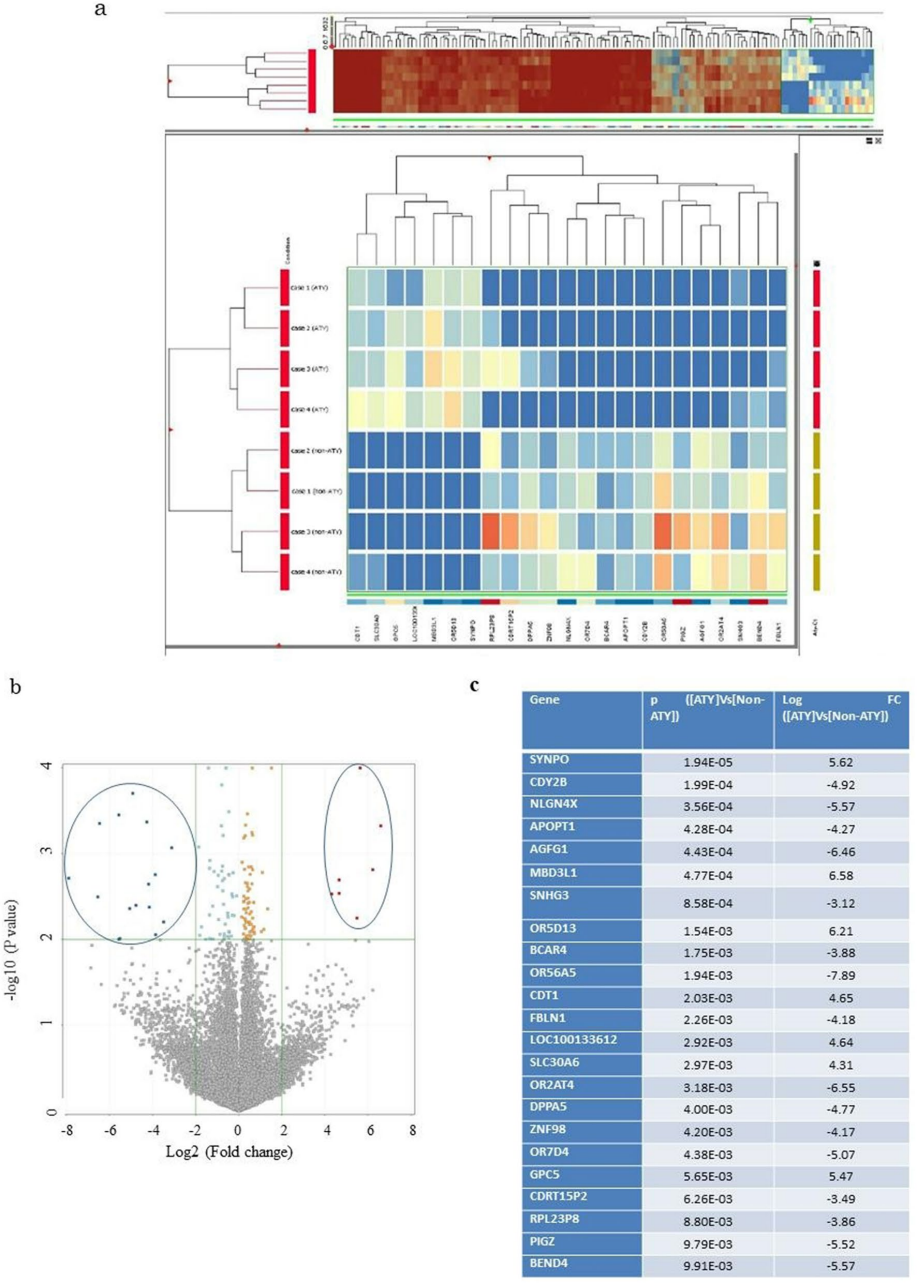
The 23 genes associated with the recurrence of hepatocellular carcinoma (HCC) were extracted using AmpliSeq transcriptome analysis in Cohort 2. (**a**) Results of clustering analysis (display of 134 genes satisfying the p value cutoff of 0.01). Data were analyzed using GeneSpring GX software (version 2.0, Agilent Technologies, Inc., Santa Clara, CA, United States). Clustering analysis showed that samples of atypical hepatocytes and nonatypical hepatocytes belonged to different groups. (**b**) Results of the volcano plot and the 23 extracted genes were analyzed using GeneSpring GX v2.0 software (Agilent Technologies). Both open circles represent selected gene groups. (**c**) Twenty-three genes that showed P values less than 0.01 and fold changes greater than 4.0 were extracted. Data were analyzed by GeneSpring GX v.2.0 software (Agilent Technologies). A bioinformatics analysis strategy for RNA-sequencing analysis was evaluated with a correction for multiplicity of statistical test according to the manufacture’s instruction. *AGFG1, ArfGAP with FG repeats 1; APOPT1, cytochrome c oxidase assembly factor 8 (COA8); BCAR4, breast cancer anti-estrogen resistance 4; BEND4, BEN domain containing 4; CDRT15P2, CMT1A duplicated region transcript 15 pseudogene 2; CDT1*, *chromatin licensing and DNA replication factor 1; CDY2B, chromodomain Y-linked 2B; DPPA5, developmental pluripotency associated 5; FBLN1, fibulin 1; non-ATY; hepatocytes other than regenerative atypical hepatocytes.; SLC30A6, solute carrier family 30 member 6; GPC5, glypican 5; LOC100133612: LOC1134 (Gene ID: 100133612), long intergenic nonprotein coding RNA 1134; NLGN4X, neuroligin 4 X-linked; MBD3L1, methyl-CpG binding domain protein 3 like 1; OR2AT4, olfactory receptor family 2 subfamily AT member 4; OR56A5, olfactory receptor family 56 subfamily A member 5; OR5D13, olfactory receptor family 5 subfamily D member 13; OR7D4, olfactory receptor family 7 subfamily D member 4; PIGZ, phosphatidylinositol glycan anchor biosynthesis class Z; RPL23P8, ribosomal protein L23 pseudogene 8; SNHG3, small nucleolar RNA host gene 3; SYNPO, synaptopodin; ZNF98, zinc finger protein 98.*

The 134 genes showing significantly different expression levels (p < 0.01) were considered to be differentially expressed genes between atypical hepatocytes and nonatypical hepatocytes (Fig. 2a) in Cohort 2. A volcano plot of the gene expression is shown in Fig. 2b. Among these 134 genes, 23 showed > fourfold change using GeneSpring GX software (version 2.0; Agilent Technologies; Fig. 2c). Figure 2b shows the volcano plot, and both open circles represent selected gene groups. Clustering analysis of the 134 genes showed that the abovementioned genes belong to the group of the rightmost 23 genes (Fig. 2a upper image) and that atypical hepatocyte and nonatypical hepatocyte populations belong to different groups. We thus assumed that the 23 genes were associated with the extent of atypical hepatocytes and investigated the signature genes related to the extent of atypical hepatocytes among these 23 genes. Figure 2c shows these 23 extracted genes.

### *CDT1* mRNA expression is strongly associated with the extent of atypical hepatocytes

We then investigated for the genes among these 23 genes that were most associated with the extent of atypical hepatocytes. The expression of each gene mRNA was quantified using the formula *ΔCt* = *Ct *_*for each gene test*_*: Ct *_*β-actin*_. The ΔCt value of all 23 genes in Cohort 3 patients (n = 62) with noncancerous frozen liver tissue samples was quantified using RT-qPCR*.* We examined the association between the ΔCt value of 23 genes and the extent of atypical hepatocytes in FFPE noncancerous HE staining sections in Cohort 3 (Supplementary Table S2). The ΔCt value of genes related to the extent of atypical hepatocytes included zinc finger protein 98 (*ZNF98*) (r = − 0.358, p = 0.0042), phosphatidylinositol glycan anchor biosynthesis class Z (*PIGZ*) (r = − 0.325, p = 0.0097), small nucleolar RNA host gene 3 (*SNHG3*) (r = − 0.333, p = 0.008), solute carrier family 30 member 6 (*SLC30A6*) (r = − 0.264, p = 0.0381), glypican 5 (*GPC5*) (r = − 0.29, p = 0.0222), cytochrome c oxidase assembly factor 8 (*APOPT1*) (r = − 0.296, p = 0.0155) and *CDT1* (r = − 0.678, p < 0.0001). The results indicated that *CDT1* ΔCt value was most associated with the extents of atypical hepatocytes (Table 2).

**Table 2:** Correlation between the extent of atypical hepatocytes and ΔCt value of the 23 genes in frozen noncancerous liver tissues in Cohort 3. (n = 62).

Gene	r	p
*CDT1*	− 0.6784	< 0.0001
*AGFG1*	− 0.2218	0.0832
*GBLN1*	− 0.1659	0.1974
*SLC30A6*	− 0.2640	0.0381
*GPC5*	− 0.2900	0.0222
*SYNPO*	− 0.2867	0.0239
*OR56A5*	0.0682	0.5983
*NLGN4X*	− 0.2442	0.0558
*OR2AT4*	− 0.3500	0.7873
*DPPA5*	0.0285	0.8257
*BEND4*	− 0.1950	0.1288
*ZNF98*	− 0.3586	0.0042
*OR5D13*	− 0.0459	0.7230
*OR7D4*	− 0.0037	0.9771
*APOPT1*	− 0.2963	0.0155
*CDY2B*	0.0762	0.5559
*PRL23P8*	− 0.0992	0.4432
*PIGZ*	− 0.3259	0.0097
*SNHG3*	− 0.3338	0.0080
*BCAR4*	− 0.1490	0.2476
*CDRT15P2*	− 0.0754	0.5603
*LOC100133612*	− 0.2407	0.0595
*MBD3LA*	− 0.0658	0.6114

ΔCt = Ct _for each of interest_ − Ct _β-actin_ Data were analyzed using the Spearman’s rank correlation test.

*AGFG1, ArfGAP with FG repeats 1; APOPT1, cytochrome c oxidase assembly factor 8 (COA8); BCAR4, breast cancer anti-estrogen resistance 4; BEND4, BEN domain containing 4; CDRT15P2, CMT1A duplicated region transcript 15 pseudogene 2; CDT1, chromatin licensing and DNA replication factor 1; CDY2B, chromodomain Y-linked 2B; DPPA5, developmental pluripotency associated 5; FBLN1, fibulin 1; LOC100133612: LOC1134 (Gene ID: 100133612), long intergenic nonprotein coding RNA 1134; NLGN4X, neuroligin 4 X-linked; MBD3L1, methyl-CpG binding domain protein 3 like 1; OR2AT4, olfactory receptor family 2 subfamily AT member 4; OR56A5, olfactory receptor family 56 subfamily A member 5; OR5D13, olfactory receptor family 5 subfamily D member 13; OR7D4, olfactory receptor family 7 subfamily D member 4; PIGZ, phosphatidylinositol glycan anchor biosynthesis class Z; RPL23P8, ribosomal protein L23 pseudogene 8; SLC30A6, solute carrier family 30 member 6; GPC5, glypican 5;SNHG3, small nucleolar RNA host gene 3; SYNPO, synaptopodin; ZNF98, zinc finger protein 98.*

### *CDT1* ΔCt value is most associated with the postoperative recurrence

We further investigated the association between *CDT1*ΔCt value and postoperative recurrence in Cohort 3. The presence or absence of postoperative recurrence and time to postoperative recurrence of HCC was independently associated with ΔCt value of *CDT1* (HR = 0.47, 95% CI 0.302–0.698, p = 0.0001), ΔCt value of breast cancer anti-estrogen resistance 4 (*BCAR4*) (HR = 0.396, 95% CI 0.220–0.734, p = 0.0028), and ΔCt value of Charcot-Marie-Tooth disease type 1A duplicated region transcript 15 pseudogene 2 (*CDRT15P2*) (HR = 4.454, 95% CI 1.619–13.026, p = 0.0035) (Table 3).

**Table 3:** Genes affecting the presence or absence of the postoperative recurrence and time to postoperative recurrence in patients with hepatocellular carcinoma in Cohort 3 by Cox multivariate proportional hazards regression model (n = 62).

Gene (ΔCt)	Hazard ratio	95% CI		p
*CDT1*	0.470	0.302	0.698	0.0001
*AGFG1*	2.200	0.443	12.756	0.3426
*FBLN1*	0.773	0.332	1.910	0.5630
*SLC30A6*	0.228	0.008	5.640	0.3665
*GPC5*	1.005	0.596	1.715	0.9861
*SYNPO*	0.574	0.158	2.043	0.3902
*OR56A5*	1.291	0.353	4.693	0.6964
*NLGN4X*	1.570	0.734	3.363	0.2413
*OR2AT4*	1.994	0.902	4.475	0.0881
*DPPA5*	0.272	0.087	0.865	0.0227
*BEND4*	2.151	0.923	4.787	0.0745
*ZNF98*	0.200	0.033	1.072	0.0604
*OR5D13*	1.523	0.997	2.202	0.0516
*OR7D4*	1.734	0.878	3.556	0.1137
*APOPT1*	0.756	0.360	1.564	0.4508
*CDY2B*	0.980	0.689	1.404	0.9095
*RPL23P8*	1.865	0.472	6.236	0.3494
*PIGZ*	1.479	0.682	3.287	0.3216
*SNHG3*	0.716	0.193	2.683	0.6163
*BCAR4*	0.396	0.200	0.734	0.0028
*CDRT15P2*	4.455	1.619	13.026	0.0035
*LOC100133612*	0.673	0.220	2.239	0.5047
*MBD3L1*	0.610	0.350	1.000	0.0502

The mRNA expression of each gene in 62 cases with noncancerous frozen liver samples was quantified using RT-qPCR*.* Data were analyzed using the Cox multivariate proportional hazards regression model.

**Figure 3:**
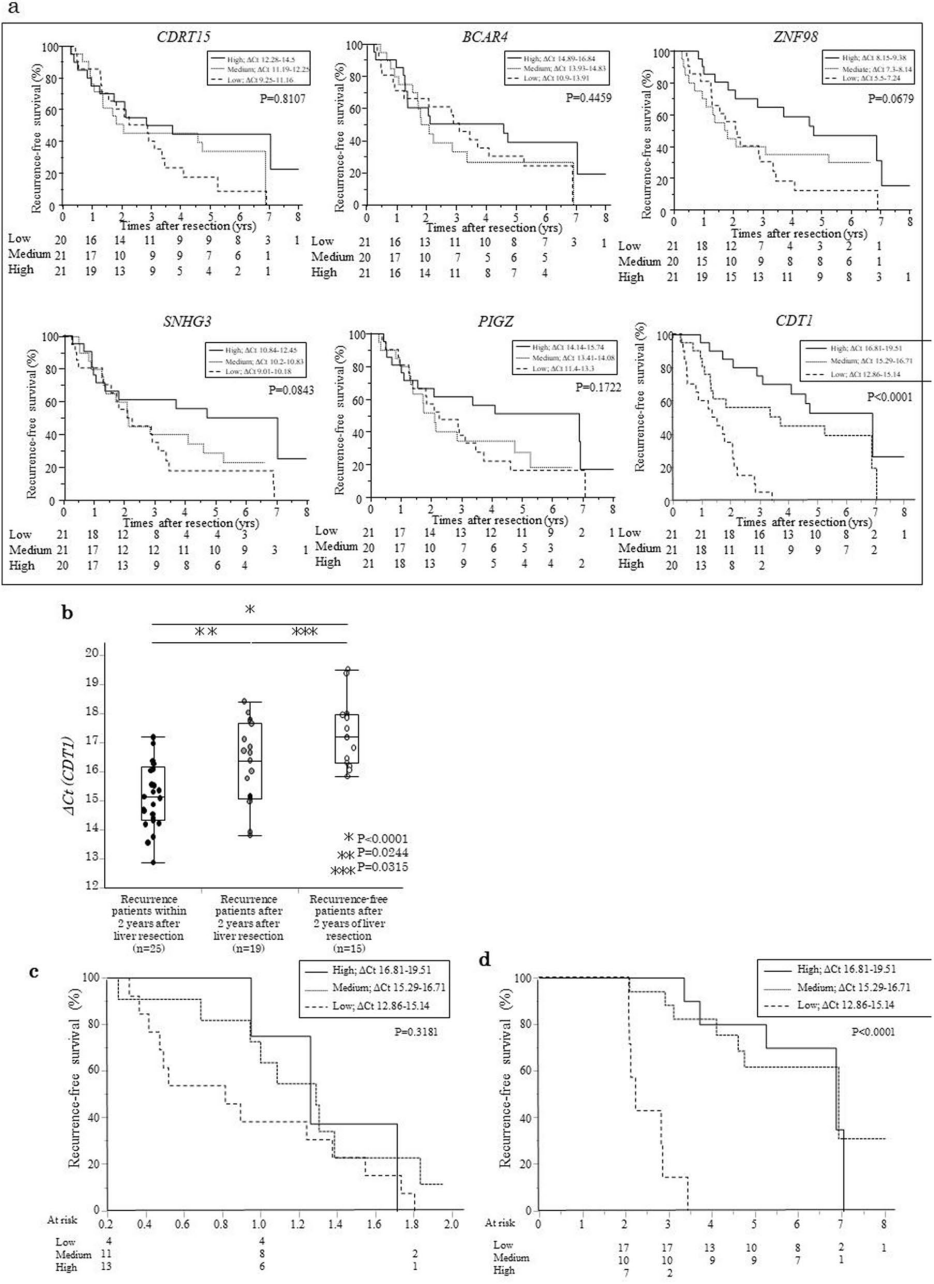
(**a**) Cumulative incidence of relapse free survival (RFS) in relation to the mRNA expression (ΔCt value) of *CDRT15P2*, *BCAR4*, *ZNF98*, *SNHG*, *PIG and CDT1* was evaluated according to three different levels of expression per gene in Cohort 3. The ΔCt value *of CDRT15P2* (p = 0.8107), *BCAR4* (p = 0.4459), *ZNF98* (p = 0.0679), *SNHG3* (p = 0.0843), *PIGZ* (p = 0.1722), and *CDT1* (high versus low: p < 0.0001). Data were analyzed using the Kaplan–Meier method, and differences among the groups were analyzed using the log-rank test. Expression was analyzed by real-time reverse transcription-quantitative PCR (RT-qPCR). Quantification was performed using the Delta (Δ)Ct method. ΔCt = Ct _for each gene of interest_: Ct _β-ACTIN_. (**b**) Comparison of ΔCt value *of CDT1* among early and late postoperative recurrence, categorized as postoperative recurrence within 2 years, equally or after 2 years, and no recurrence after 2 years in Cohort 3. There was a significant difference in ΔCt value *of CTD1* between patients with recurrence within 2 years after liver resection and equally or after 2 years (p = 0.0244), patients with postoperative recurrence-free patients after 2 years and equally or after 2 years (p = 0.0315) and patients with recurrence within 2 years (p < 0.0001). Data were analyzed using the Kruskal–Wallis and Steel–Dwass tests. (**c**) The cumulative incidence of RFS was compared according to three different groups of ΔCt value *of CDT1* in patients with early postoperative recurrence in Cohort 3. RFS was not significantly different among the three groups (p = 0.3181). (**d**) The cumulative incidence of RFS was compared according to three different groups of ΔCt value *of CDT1* in patients with late postoperative recurrence in Cohort 3. RFS was significantly higher in the high ΔCt group than in the low ΔCt value group (high versus low: p < 0.0001).

Then, we verified the association between postoperative recurrence time and the ΔCt value of *CDT1* by the cumulative incidence of RFS in Cohort 3 (n = 62). The cumulative incidence of RFS was compared based on the ΔCt value of *CDT1*, classified into three groups (high, medium, and low expression), to determine its predictive value. At 5 years, RFS was 0% (HR = 1.0, reference), 48.6% (HR = 0.216, 95% CI 0.08–0.489, p = 0.0002), and 49.8% (HR = 0.145, 95% CI 0.055–0.342, p < 0.0001) in the high, medium, and low ΔCt value groups, respectively (Fig. 3a). At 5 years, RFS was significantly lower in the low ΔCt value group than in the high or medium ΔCt value group in CDT1.

Furthermore, the ΔCt values of *ZNF98*, *PIGZ*, and *SNHG3*, which were significantly associated with extent of atypical hepatocyte, and *BCAR4*, and *CDRT15P2*, which were significantly associated with postoperative recurrence time, were divided into three groups according to the ΔCt values and each RFS was compared. The results showed no significant difference in RFS among the three groups for any of the genes (Fig. 3a). Notably, even if cumulative RFS was compared by evenly dividing the ΔCt value into three groups from the minimum ΔCt value, there was a significant difference, indicating that ΔCt value of *CDT1* and RFS were significantly associated.

### Association between ΔCt value of *CDT1* and early- and late- postoperative recurrence

We examined the association between ΔCt value of *CDT1* and *early- and late-*recurrence times, categorized as postoperative recurrence within 2 years (early), equal to or after 2 years, and no recurrence after 2 years (late) in Cohort 3. The ΔCt value of *CDT1* was lower in the early-recurrence group than in the late-recurrence groups (early versus (vs.) late; p = 0.0244, early vs. no recurrence; p < 0.0001, early vs. no recurrence; p = 0.0315, Fig. 3b).

Next, patients with early postoperative recurrence showed no difference in RFS among the three ΔCt value of *CDT1*groups (p = 0.3181, Fig. 3c). In patients with late postoperative recurrence, RFS was significantly higher in the high ΔCt value group than in the low ΔCt value group (p < 0.00001, Fig. 3d). These data indicated that expression of *CDT1* gene was more associated with late recurrence. Therefore, *CDT1* gene expression was found to correlate the most strongly with postoperative recurrence time among the 23 genes.

### Cells strongly expressing CDT1 are more similar to atypical hepatocytes

**Figure 4:**
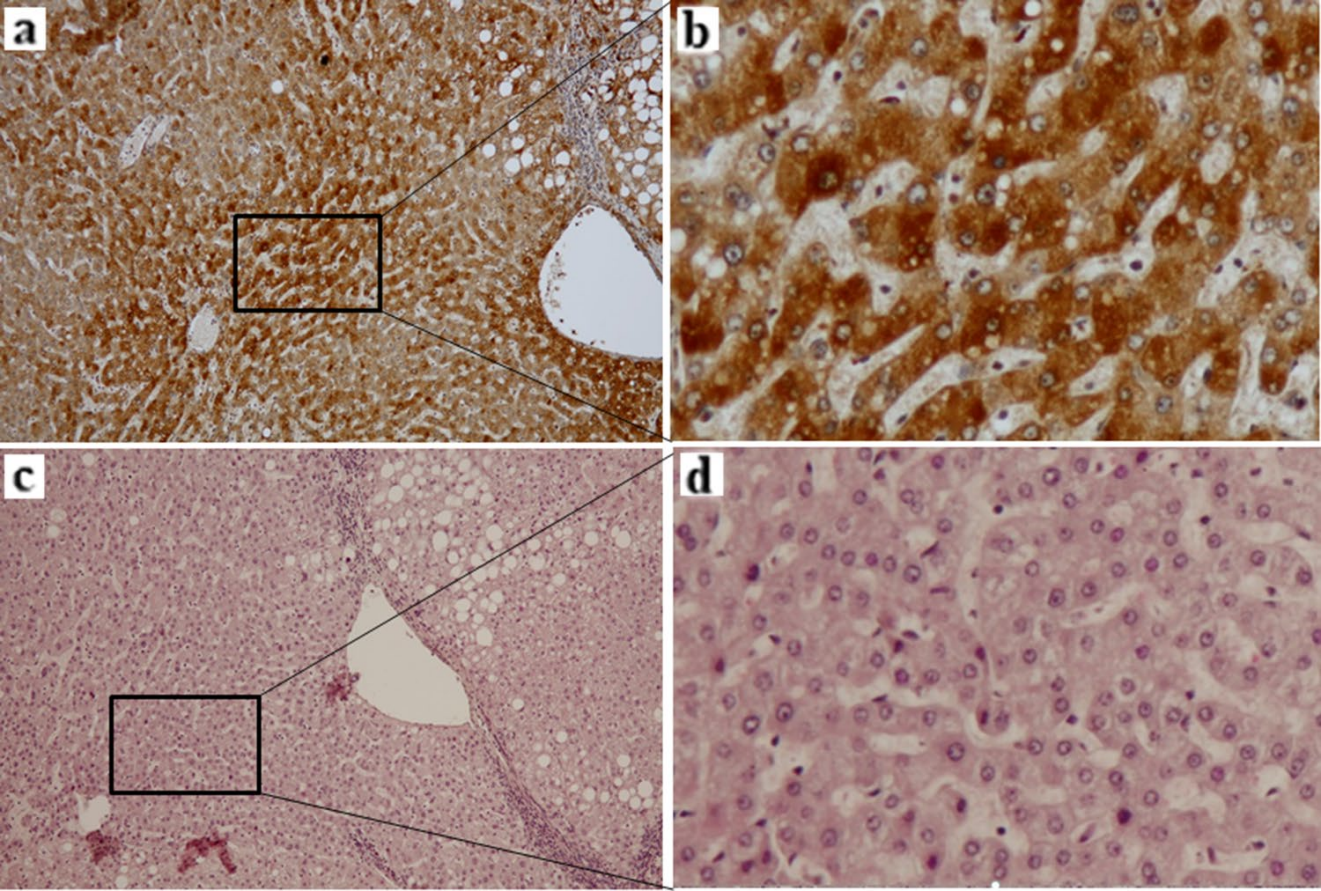
Immunohistochemistry revealed that CDT1-positive cells corresponded to atypical hepatocytes. (**a**) Image show lower magnification of CDT1 staining. (Counterstained by hematoxylin, × 100). (**b**) The area circled by the square indicates CDT1-positive cell aggregation. (Counterstained by hematoxylin, × 400). (**c**) Image of HE staining shows almost the same section as the CDT1-staining image of (**a**) (HE, × 100). (**d**) The area circled by the square indicates atypical hepatocytes aggregation (HE, × 400).

**Figure 5:**
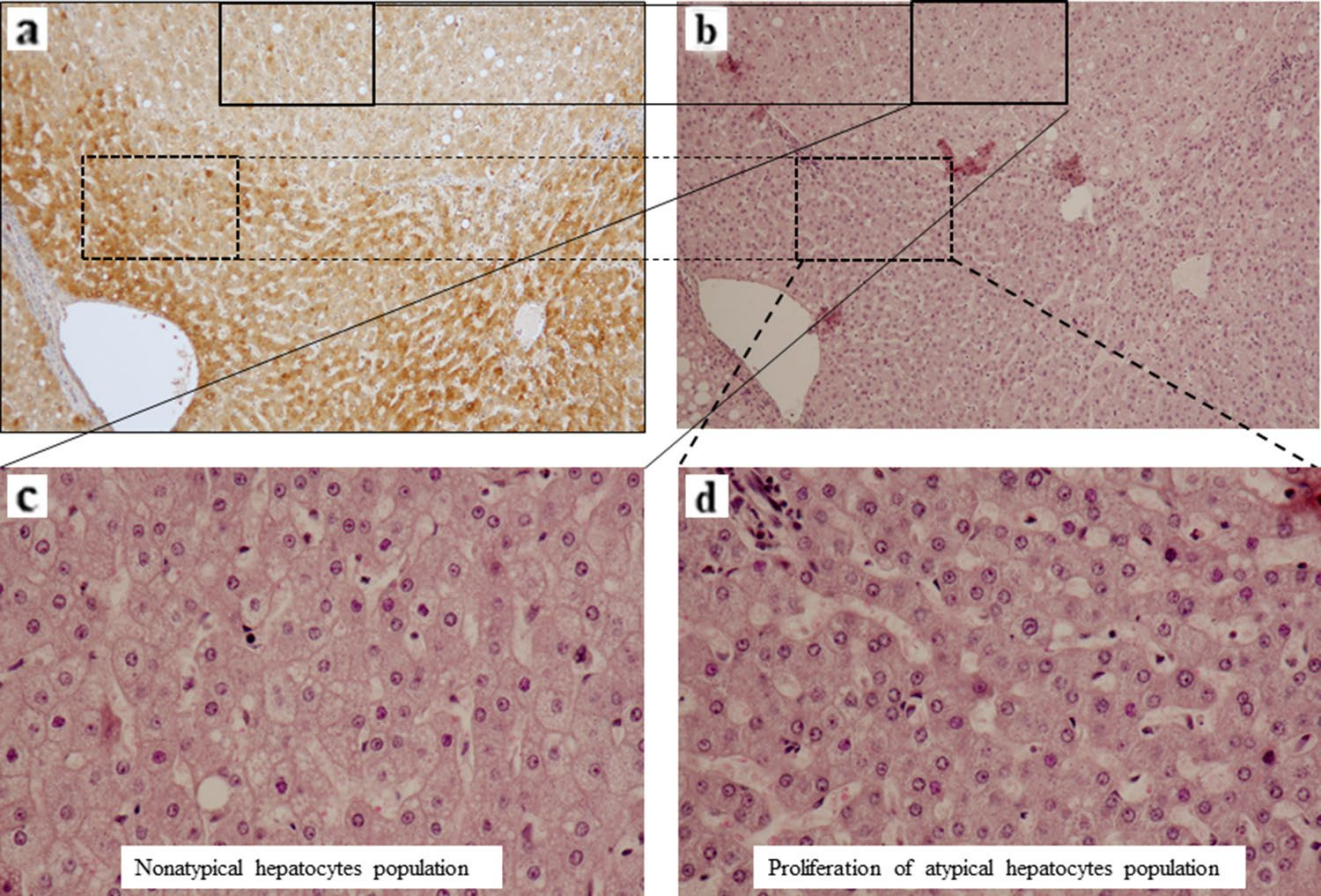
Comparison of CDT1 staining cells and hematoxylin and eosin (HE) staining cells in almost the same area. (**a**) Images showing CDT1 staining (counterstained by hematoxylin, × 100). The square along the line indicates a cell population that is negative or weak for CDT1 staining. (**b**) Image of HE staining shows almost the same area as the CDT1-staining image of (a) (HE, × 100) of continuous sections. The dashed line along the line on the HE staining image indicates a positive cell population for CDT1 staining. (**c**) A higher magnification image of the square of (**a**) and (**b**) is presented (HE, × 400). Nonatypical hepatocytes population were observed. (**d**) CDT1 staining was consistently found in cells morphologically considered atypical hepatocytes (HE, × 400).

The differences in CDT1 expression in FFPE noncancerous liver sections in Cohort 4 observed using IHC are shown in Fig. 4a,b. CDT1 staining images (Fig. 4a) and HE staining images (Fig. 4c) of the continuous section of noncancerous liver of the same patient are presented. CDT1-positive cells appeared in a mosaic formation in lobules (Fig. 4a). The CDT1-positive cells that exhibited stronger expression resembled those morphologically considered to be atypical hepatocytes (Fig. 4b,d). Importantly, the stainability of CDT1 was clearly distinguished between atypical hepatocytes and nonatypical hepatocytes, even in the same lobules. Different CDT1 expression levels were observed among the atypical hepatocytes populations, and even in hepatocytes other than atypical hepatocytes, the stainability of CDT1-positive cells varied (Figs. 4a,b, 5a).

Figure 5a,b showed comparison between of CDT1 staining cells and HE staining cells in almost the same area. The square along the line indicates a cell population that is negative or weak for CDT1 staining (Fig. 5c), and the square along the dashed line indicates a cell population that is strong for CDT1 staining (Fig. 5d). CDT1 staining was consistently found in cells morphologically considered atypical hepatocytes. The five different hepatocyte groups of the IR of hepatocytes showed weaker staining levels than the atypical hepatocytes. In addition, CDT1 staining was observed in lymphocytes that infiltrated the portal area and in necro-inflammatory reaction areas. Based on the above results, we confirmed that CDT1 expression is a distinctive indicator of the extent of atypical hepatocytes.

**Figure 6:**
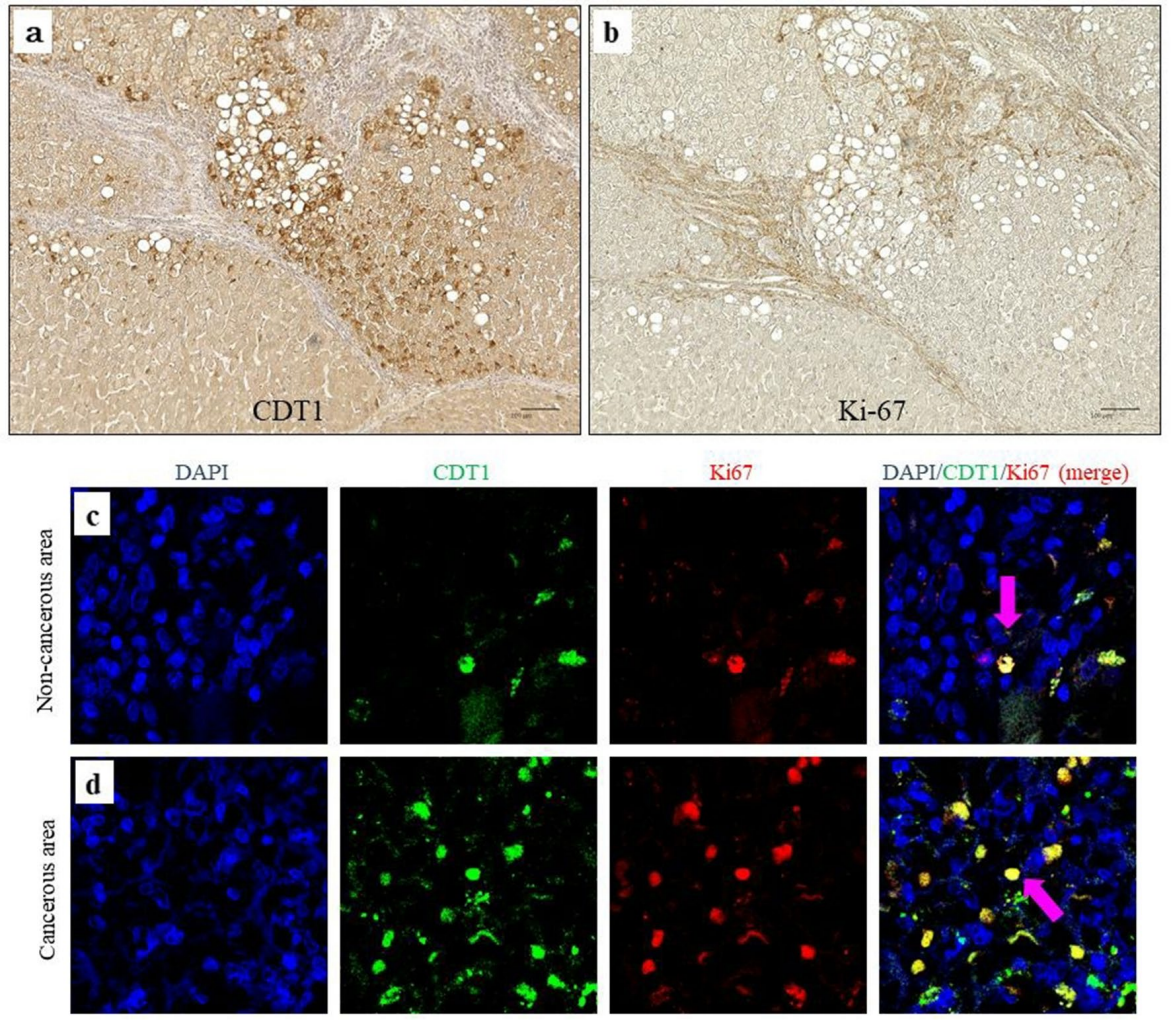
Comparison of Immunohistochemistry images of CDT1-positive and Ki-67-positive hepatocytes in lobules in continuous noncancerous formalin-fixed paraffin-embedded (FFPE) noncancerous liver sections in Cohort 4. (**a**) Localization of CDT1-positive cells in lobules (counterstained with hematoxylin, × 100). (**b**) Localization of Ki-67-positive cells in lobules (counterstained with hematoxylin, × 100). (**c**) Representative immunofluorescence images of CDT1, Ki-67 and Prolong, which contained 4',6-diamidino-2-phenylindole (DAPI), in noncancerous FFPE liver sections. Blue, nuclei counterstained with Hoechst 33342, green, CDT1, red, Ki-67 and merged image (right). Arrows indicate the colocalization of CDT1 with Ki-67 in the nuclei of hepatocytes. (**d**) Representative immunofluorescence images of CDT1, Ki-67 and DAPI in cancerous FFPE liver sections. Blue, nuclei counterstained with DAPI, green, CDT1, red, Ki-67 and merged image. Arrows indicate the colocalization of CDT1 with Ki-67 in the nuclei of cancer cells.

Next, we compared the localization of Ki-67- and CDT1-positive cells within the same section in Cohort 4. CDT1-positive (Fig. 6a) and Ki-67-positive hepatocytes (Fig. 6b) were scattered in the parenchyma. Ki-67-positive cells were observed mainly in the nucleus and were scattered in lobules. Furthermore, the CDT1 staining pattern in noncancerous sections was scattered and more extensive than that of Ki-67-positive cells. We observed atypical hepatocytes and cancer cells that were positive for both Ki-67 and CDT1 (Fig. 6c,d) by CLSM. The merged images revealed colocalization of CDT1 and Ki-67, mainly in the nuclei of hepatocytes and cancer cells, representing 360° view movies of 3D reconstructed images of CDT1- and Ki-67-positive cells. It was confirmed that Ki-67 and CDT1 colocalized in the same nucleus in the cancer cells and hepatocytes in lobule (Supplementary Fig. S5; 3D view image).

### Comparison of ΔCt value of *CDT1* among nonatypical hepatocytes, atypical hepatocytes and cancer cells

**Figure 7:**
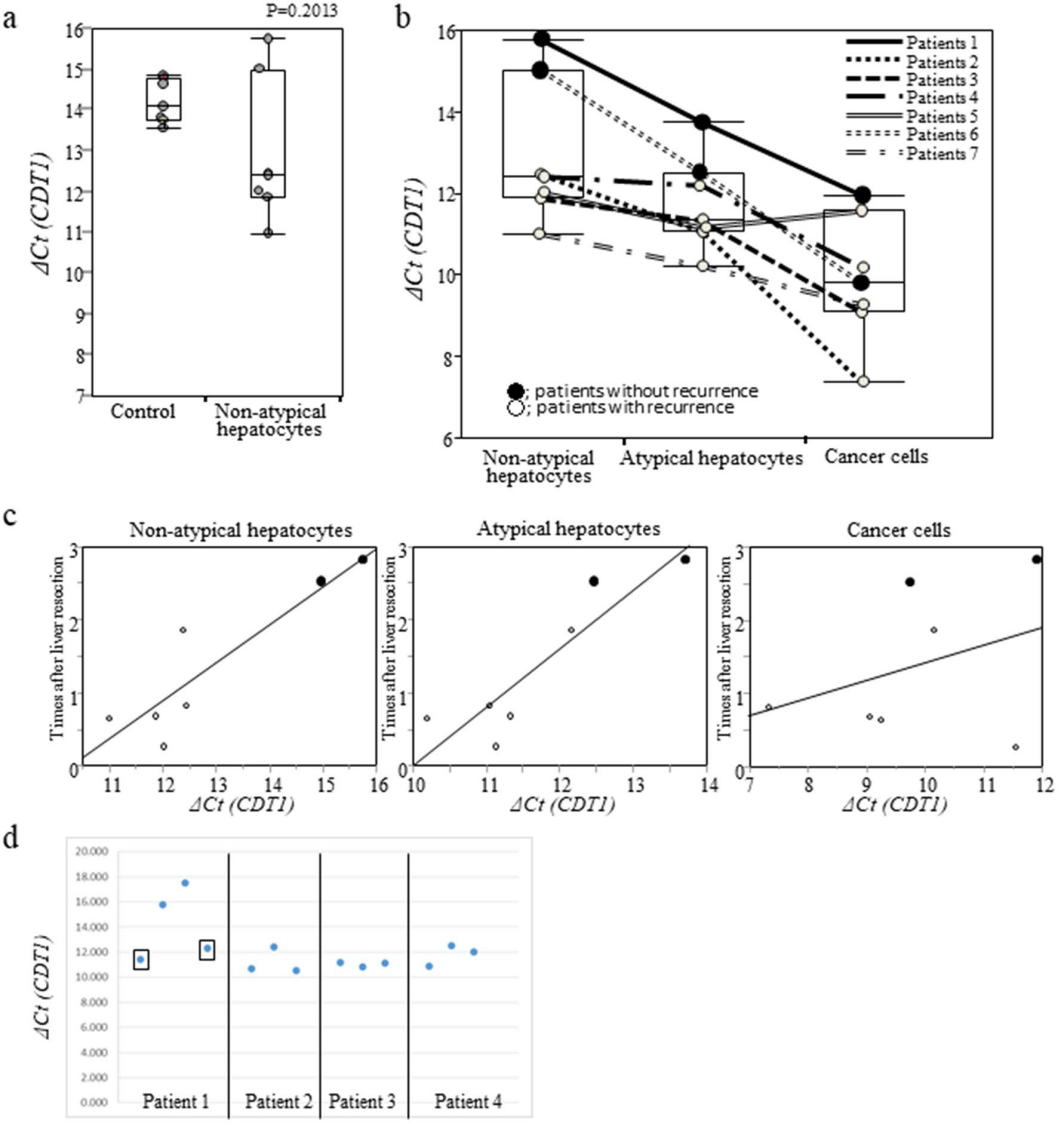
Comparison of ΔCt value *of CDT1* at different hepatocytes populations in formalin-fixed paraffin-embedded (FFPE) tissues in Cohort 5. (**a**) The panel shows ΔCt value *of CDT1* of the nonatypical hepatocyte population and that of the controls. Data were analyzed by Kruskal–Wallis tests. (**b**) The panel shows the distribution of ΔCt value *of CDT1* in the nonatypical hepatocyte population, the atypical hepatocyte population and in cancer cells. Closed circles represent patients did not develop recurrence, and the others represent patients who became recurrence. (**c**) The relationship among ΔCt value *of CDT1* and duration of observation in the nonatypical hepatocyte population, the atypical hepatocyte population, and cancer cells and time to recurrence. Closed circles represent nonrecurrent patients, and open circles represent recurrent patients. Data were analyzed by Spearman’s rank correlation test. (**d**) Comparison of the ΔCt value *of CDT1* in the nonatypical hepatocyte population (closed circle) or similar atypical hepatocytes (squared circle) at multiple populations from the same section. Data were analyzed by the Kruskal–Wallis test and Spearman's rank correlation test.

We examined differences in ΔCt value of *CDT1* among the three population in nonatypical hepatocytes, atypical hepatocytes and cancer cells in the same FFPE sections in different cell populations in Cohort 5 by LCM. The ΔCt value of *CDT1* in the control liver section tended to be higher than that in the noncancerous liver section in the nonatypical hepatocytes population (p = 0.2013, Fig. 7a). Those showing a greater grade or a strong degree of atypical hepatocytes tended to have lower ΔCt value of *CDT1* in the nonatypical hepatocytes (Fig. 7b). Some of the nonatypical hepatocytes had a low ΔCt value of *CDT1*, indicating that some of the nonatypical hepatocytes were not usual regenerative hepatocytes. These cells were considered to be rather close to atypical hepatocytes. In addition, even among the nonatypical hepatocytes, there was variability in *CDT1* expression based on morphological variation.

Furthermore, it was suggested that those who showed high ΔCt value of *CDT1* in the nonatypical hepatocytes were less likely to relapse than those with low ΔCt value (Fig. 7b). A representative patient is shown in Fig. 7b. Patient 1, in whom most of the hepatocytes in noncancerous sections appeared to be morphologically close to usual regenerative hepatocytes, had a high ΔCt value of *CDT1*, and no recurrence was observed during the observation time (closed circles show nonrecurrence, and open circles show patients with recurrence; Fig. 7b). When the relationships among ΔCt value of *CDT1* in the nonatypical hepatocytes, the atypical hepatocytes, and cancer cells and times to recurrence were represented, there was a significant correlation between the ΔCt value of *CDT1* in nonatypical hepatocytes (r = 0.898, p = 0.006) and atypical hepatocytes (r = 0.905, p = 0.0051) and the times to recurrence (Fig. 7c). Figure 7d shows the ΔCt value of *CDT1* in the nonatypical hepatocytes (closed circle), near (close) the atypical hepatocytes (squared circle), and in the nonatypical hepatocytes from the same patient. Even in the same patient, differences in ΔCt value of *CDT1* were observed depending on the area and cell morphology.

**Figure 8:**
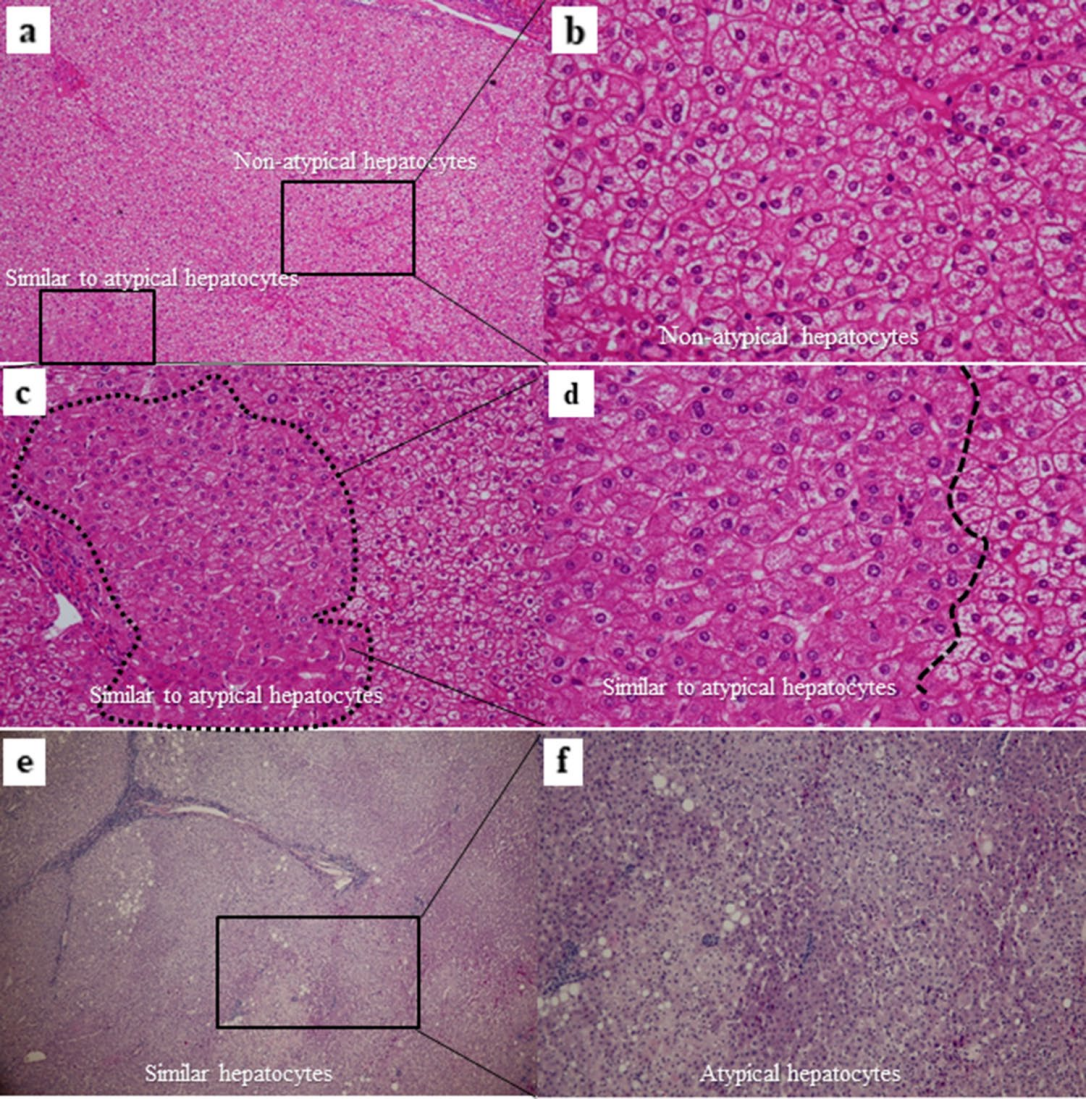
Representative examples (Patients No. 1 and No. 2 in Fig. 7b) of atypical hepatocytes and nonatypical hepatocytes populations in Cohort 5 that were collected by laser capture microdissection (LCM). (**a**) Image represents a lower magnification of a section of patient 1 in Fig. 7b (Hematoxylin and eosin staining (HE), × 40). (**b**) Image represents a higher magnification of a square in a nonatypical hepatocyte population of (**a**) that was morphologically close to usual regenerative hepatocytes (HE, × 200). (**c**) Image represents a square of the hepatocytes population in (**a**) close to the atypical hepatocyte (dashed line; HE, × 100). (**d**) Image represents a higher magnification of the dashed line area in (**c**) (HE, × 200). (**e**) Image represents a lower magnification in patient No. 2 of Fig. 7b (HE, × 40). (**f**) Image represents a higher magnification of a square of (**e**) (HE, × 100).

The images of Fig. 8a–f showed a noncancerous liver HE-staining section in patients No. 1 (Fig. 8a–d) and No. 2 (Fig. 8e,f) in Cohort 5. The hepatocytes represented are close to usual regenerative hepatocytes in Fig. 8a,b. On the other hands, images of Fig. 8c,d showed the hepatocytes represented are close to atypical hepatocytes. These hepatocytes populations were collected from serial sections from Patient 1. A greater extent of atypical hepatocytes was observed in the noncancerous section of Patient 2 in Fig. 8e. Various mosaic-like morphological changes in hepatocytes were observed in higher magnification of Fig. 8e (Fig. 8f). Even some patients that presented such histological findings with nonatypical hepatocytes tended to have low ΔCt value of *CDT1* and had recurrence shortly after resection.

### CDT1 knockdown inhibited Huh7 cell proliferation

**Figure 9:**
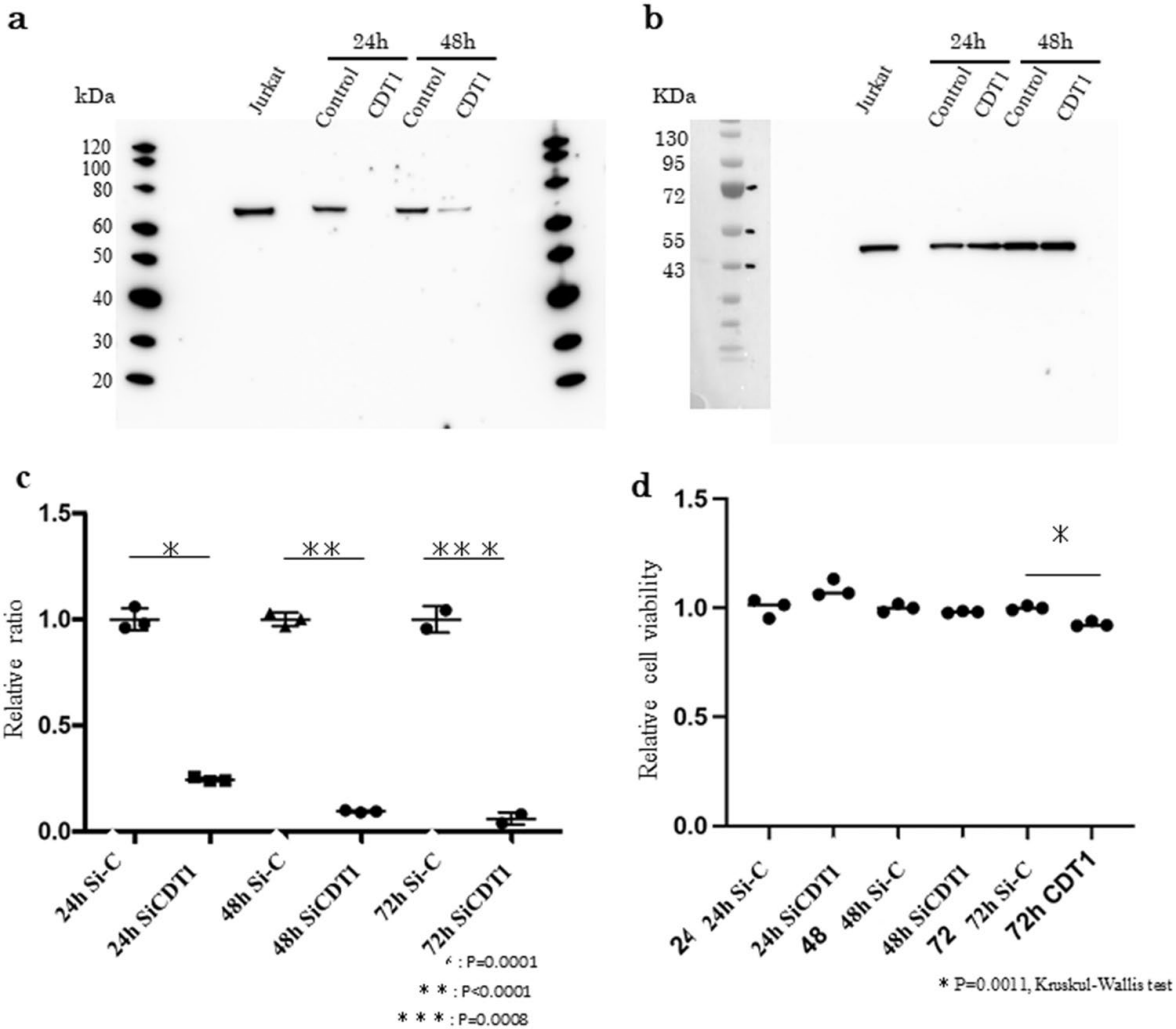
Representative images of Western blotting analysis of CDT1 siRNA (SiCDT1)- and control siRNA (Si–C)-transfected Huh7 cells. (**a**) Representative images of CDT1 expression in 24-h and 48-h incubated SiC- and SiCDT1-transfected Huh7 cells. Jurkat cells are a positive control. (**b**) Representative images of β-actin expression at 24 h and 48 h in the SiCDT1- and Si–C-transfected Huh7 cells. (**c**) Comparison of ΔCt value *of CDT1* at 24 h, 48 h and 72 h between the Huh7 cells transfected with SiCDT1 and Si–C. (*; p = 0.0001, **; p < 0.0001, ***; p = 0.0008) Data were analyzed by the Kruskal–Wallis test. (**d**) Comparison of the results of the 3-(4,5-dimethylthiazol-2yl)-2,5-diphenyltetrazolium bromide (MTT) assay at 24 h, 48 h and 72 h using the SiCDT1 and Si–C in Huh7 cells. (*; p = 0.0011). Data were analyzed by Kruskal–Wallis tests.

We evaluated the function of CDT1 in vitro using Huh7 cells. After CDT1 siRNA (Si-CDT1) transfection, the protein expression of CDT1 was clearly reduced compared to that of the control siRNA (Si–C) group at 24 h, 48 h and 72 h after transfection (Fig. 9a). Figure 9b shows β-actin in the siRNA-transfected Huh7 cells. The original blots/gels are presented in Supplementary Figs. S6, S7, S8 and S9.

Comparison of the levels of ΔCt value of *CDT1* between the Huh7 cells transfected with SiCDT1 and control Si–C showed that ΔCt value of *CDT1* was expressed at significantly lower levels in the SiCDT1-transfected cells than in the Si–C-transfected cells (p = 0.0495, Fig. 9c). Then, we performed MTT assays using the siRNA-transfected Huh7 cells or control Huh7 cells. CDT1 siRNA transfection significantly inhibited the proliferation of Huh7 cells (p = 0.0495, Fig. 9d).

## Discussion

We found that the extent of atypical hepatocytes was the most significant risk factor for HCC postoperative recurrence. Furthermore, the genes most associated with the extent of atypical hepatocytes were consistent in the verification study using noncancerous liver tissues. Based on these results, we confirmed that *CDT1* is a signature gene for atypical hepatocytes. Therefore, *CDT1* expression and the extent of atypical hepatocytes in liver tissue could be histological descriptions that indicate a high carcinogenic state in the liver. In addition, these results corroborated the findings of our previous study, which showed that the extent of atypical hepatocytes was an important background risk factor for the occurrence of HCC in patients with chronic hepatitis C or LC^13^.

As hepatocytes constantly undergo necrosis and regeneration during chronic liver diseases, unusual regenerative hepatocytes with various genetic abnormalities may emerge. Therefore, the phenotypic traits of new regenerative hepatocytes may vary. We presume that these newly regenerated hepatocytes will progress to atypical hepatocytes. Immunohistochemical analysis demonstrated that atypical hepatocytes expressed CDT1 at the protein level. Different CDT1 expression levels were observed among the atypical hepatocytes population, and even in hepatocytes other than atypical hepatocytes, the stainability of CDT1-positive cells varied. This observation suggested that some hepatocytes already overexpressed CDT1, even though these cells were morphologically close to normal hepatocytes. This finding was consistent with our assumption that carcinogenesis occurs in a multistep process. Furthermore, based on comparisons of *CDT1* ΔCt among nonatypical hepatocytes, atypical hepatocytes and cancer cells in the same FFPE section, even within the same lobule, *CDT1* expression was heterogeneous. Thus, in those who had a mosaic pattern of CDT1 expression, the difference in ΔCt value of *CDT1* between the nonatypical hepatocytes and atypical hepatocytes was small. This finding also indicates that even morphologically usual regenerative hepatocytes have already experienced genetic abnormalities. As mentioned above, we consider that the extent of atypical hepatocytes and levels of CDT1 expression are histological indicators of a high carcinogenic state or genetic abnormalities. Moreover, in the noncancerous liver of HCC patients who showed HCV elimination (Patients No.1 in Fig. 8a), the hepatocytes other than atypical hepatocytes were morphologically uniform, and *CDT1* mRNA was weakly expressed. As these, *CDT1* mRNA was more strongly expressed in atypical hepatocytes presented in the same section than in nonatypical hepatocytes, and a clear difference was observed between the two groups. This fact also supports our rationale.

The atypical hepatocytes were identified as the hepatocyte population associated with the HCC recurrence, but semi-quantification of atypical hepatocytes in HE staining section is not robust due to intra- and inter-observer variability.　For this reason, it is essential to determine the gene expression representing the abundance of atypical hepatocytes in noncancerous liver tissue. The significance of this paper lies in the fact that we were able to establish that the expression of CDT1 is associated with the extent of atypical hepatocytes.

Next, we performed CLSM analysis of CDT1 and Ki-67. The CDT1-positive and Ki-67-positive products were colocalized in the nuclei of hepatocytes, suggesting that CDT1-positive cells, which are thought to be identical to atypical hepatocytes, may have proliferative efficacy in lobules. Similarly, both genes colocalized in the nuclei of cancer cells. Moreover, the expression of CDT1 was observed in both the nuclei of cancer cells and in the nuclei of hepatocytes in noncancerous parenchyma. However, although the number of positive cells in these noncancerous hepatocytes was lower than in cancer cells, the expression of CDT1 in the nuclei was morphologically similar. Positivity rate for Ki-67 (labeling index), a cell proliferation marker, has been reported in various cancer tissues (32–37), but several studies have indicated that Ki-67 labeling index is not a poor prognostic factor in HCC (38–40). Therefore, it has not been established as a prognostic marker for HCC.

In our results, CDT1 and Ki-67 were colocalized in some HCC cells. In addition, a comparison of the atypical hepatocyte population and HCC cells showed that CDT1-positive cells were more abundant in HCC cells and were colocalized with Ki-67. However, in morphologically normal hepatocytes, CDT1-positive cells were rarely observed. Thus, the fact that Ki-67 and CDT1 colocalize in some hepatocytes and cancer cells nuclei suggests that CDT1 may be involved in active cell proliferation. We consider that this gene may be upregulated in atypical and cancer cells which includes active cell cycles.

CDT1 expression is commonly upregulated in G1 phase and is reduced in S phase^41–43^. There have been several reports on the relationship of *CDT1* with carcinogenesis and tumor development^44–46^. Furthermore, CDT1 expression was shown to be upregulated in HCC tissues. Yu et al.^47^ reported that CDT1 was coexpressed with α-fetoprotein in HCC tissues. Karavias et al.^44^ reported that high CDT1 expression was correlated with reduced overall survival. These findings support the current results. Hu et al.^48^ reported that abnormalities in geminin or minichromosome maintenance (MCM) 2–7 lead to the overexpression of *CDT1*. Recently, Cai et al. stated that the MCM family controls *CDT1* expression, and overexpression of *CDT1* promoted the growth of cancer cells in vitro^49^. Coulombe et al.^50^ demonstrated that chromosome 13 open reading frame 7/ring finger protein 219 (*C13ORF7/RNF219)* controlled *CDT1* expression. However, our transcriptome analysis did not reveal differences in the expression of these genes between atypical hepatocytes and nonatypical hepatocytes.

Herein, we could not confirm the exact reasons for the observed *CDT1* overexpression, which warrants further investigation. However, we consider it essential to investigate the possibility of malignant transformation by overexpression of *CDT1* in human hepatocyte cell lines rather than experimental systems using cancer cell lines, as reported previously. To confirm that atypical hepatocytes are a preneoplastic entity implicated in the multistep carcinogenesis process, we must also verify whether any oncogene alterations are observed in these cells and determine how gene expression differs between them and cancer cells in the same patient liver section. Therefore, considering the heterogeneous localization of CDT1 in lobules, we will determine the presence and degree of genetic abnormalities and instability in various hepatocytes or cancer cells within lobules by applying novel spatial transcriptomics technology for visualization using FFPE tissue.

This study has several limitations. First, it is impossible to clinically diagnose whether recurrence is due to multicentric origin or intrahepatic metastasis without sequencing analysis^51^. Therefore, it is not clear whether atypical hepatocytes or CDT1 expression correlated with hepatocarcinogenesis or metastasis. Second, *CDT1* mRNA expression is dependent on the number of infiltrating lymphocytes in the tissue, so to evaluate pure *CDT1* mRNA expression, it is necessary to at least exclude lymphocytes that are strongly infiltrating the portal area or cancerous tissue. We believe that our study using LCM to collect tissues without lymphocytic infiltration areas shows the true level of *CDT1* mRNA expression in tissue. Our study using LCM and tissue resection excluding lymphocytic infiltrate shows true CDT1 expression. We will continue the study with a larger independent patient cohort in the future. Next, the abovementioned methods are needed to confirm that atypical hepatocytes are a preneoplastic entity involved in a multistep carcinogenic process.

In terms of clinical significance, the grade of atypical hepatocytes is a risk factor not only for hepatocarcinogenesis but also for recurrence.

For example, we consider that if there are lot of closely-aggregated clusters of atypical hepatocytes around the cancer nodule, the prognosis of patients may be improved by enlarging the resection margin.

Therefore, postoperative follow-up can be considered for patients with a high risk of postoperative recurrence. Furthermore, preneoplastic lesions may also be targeted for prevention. Ultimately, the current findings establish a theoretical basis for improving the RFS of patients with HCC who undergo liver resection. We further emphasize that it is possible to selectively collect specific cell populations using FFPE sections and perform RNA-seq on these cell populations using NGS.

In this study, we searched for a hepatocyte population associated with postoperative recurrence and then performed RNA-seq using NGS on the specific hepatocyte population collected by LCM. Thus, it was possible to identify the signature genes involved in the cell population of the putative preneoplastic lesion. This theory is also applicable to other cancers and can be applied to detect specific regions and signature genes involved in tumor invasion and development. We propose that this will contribute to the prevention and improvement of prognosis in refractory carcinomas and will have a significant clinical contribution. Moreover, Atypical hepatocytes were identified as a hepatocyte population that is involved in recurrence, but it takes skill to use the proliferation of atypical hepatocytes as a morphological indicator of recurrence. For this reason, it is important to determine a gene that represents the abundance of atypical hepatocytes in noncancerous liver tissue.

The significance of this paper lies in the fact that we were able to establish that the expression of CDT1 is associated with the extent of atypical hepatocytes.

The fact that the CDT1 gene was identified as a marker of atypical hepatocyte abundance is also important because the high carcinogenic state can be estimated by RT-qPCR analysis of noncancerous liver tissue. From a clinical standpoint, the development of such a system would be of great significance because it would standardize the results. Another significant clinical feedback of this paper is that it enables the prediction of HCC recurrence without relying on the reading of a skilled pathologist.

The more role of CDT1 in hepato-carcinogenesis is a subject for future study. In order to investigate the genetic and epigenetic alterations for *CDT1* gene upregulation, we searched the sequence data on 160 HCC samples^52^. Exome sequence showed that *CDT1* gene harbored no mutation, and copy number gains were not found near *CDT1* gene (16q24.3). RNA sequence demonstrated that there was no fusion event of *CDT1* gene. We found that methylation status was not different between HCC and the surrounded liver samples by methylation profiling using HumanMethylation450 BeadChip (Illumina). Thus molecular mechanism for *CDT1* gene upregulation is unknown at present. Given that *CDT1* is involved in the formation of the pre-replication complex that is necessary for DNA replication, we assumed that this gene may be upregulated in atypical and cancer cells which includes active cell cycles.

In summary, we found that the extent of atypical hepatocytes are associated with the time to postoperative HCC recurrence. The expression of CDT1 is a genetic indicator of the extent of atypical hepatocytes. Furthermore, the extent of atypical hepatocytes represents a high carcinogenic state of the liver, and atypical hepatocytes could be one of the preneoplastic entities, while CDT1 seems to be essentially associated with the extent of atypical hepatocytes. Therefore, identification of the gene pathways that regulate the high expression of CDT1 provides a strategy to improve the prognosis of HCC. Furthermore, even without a skilled pathologist, we can predict the risk of HCC recurrence by measuring *CDT1* mRNA in noncancerous liver tissue.

## Supplementary Information

Supplementary Information 1.

Supplementary Information 2.

## Data Availability

All sequence reads were registered in Gene Expression Omnibus (https://www.ncbi.nlm.nih.gov/geo) under accession numbers GSE153742 and GSM4650405-GSM4650412.
