# Supplementary material for: The proliferation of atypical hepatocytes and CDT1 expression in noncancerous tissue are associated with the postoperative recurrence of hepatocellular carcinoma
Source: Sci Rep. 2022 Nov 28;12:20508. doi: 10.1038/s41598-022-25201-6 (PMC9705552; doi:10.1038/s41598-022-25201-6)
Supplement: Supplementary file 1 — Supplementary Information 1. [file 41598_2022_25201_MOESM1_ESM.pdf]

Supplementary information

The proliferation of atypical hepatocytes and CDT1 expression in noncancerous tissue are associated with the postoperative recurrence of hepatocellular carcinoma

Mitsuhiko Moriyama[1]*, Tatsuo Kanda[1]*, Yutaka Midorikawa[1], Hiroshi Matsumura[1], Ryota Masuzaki[1], Hitomi Nakamura[1], Masahiro Ogawa[1], Shunichi Matsuoka[1], Toshikatu Shibata[1], Motomi Yamazaki[1], Kazumichi Kuroda[1], Hisashi Nakayama[2] , Tokio Higaki[2], Kazunori Kanemaru[3], Toshio Miki[3], Masahiko Sugitani[4], Tadatoshi Takayama[2]

[1]Division of Gastroenterology and Hepatology, Department of Medicine, Nihon University School of Medicine, 30-1 Oyaguchi-Kamimachi, Itabashi-ku, Tokyo 173-8610, Japan

[2]Department of Digestive Surgery, Nihon University School of Medicine, 30-1 Oyaguchi-Kamimachi, Itabashi-ku, Tokyo 173-8610, Japan.

[3]Department of physiology, Division of Biomedical Sciences, Nihon University School of Medicine, 30-1 Oyaguchi-Kamimachi, Itabashi-ku, Tokyo 173-8610, Japan.

[4]Department of Pathology, Nihon University School of Medicine, 30-1 Oyaguchi-Kamimachi, Itabashi-ku, Tokyo 173-8610, Japan.

Supplementary Tables

Table S1 Clinical characteristics of patients with hepatocellular carcinoma included in Cohort 1 (n = 356)

Parameter	HBV (+)	HCV (+)	NBNC	P-values
No. of patients	50	219	87	
Age (years)	58.7 ± 10.0	68.8 ± 7.1	68.6 ± 9.5	<0.0001
Sex (male, %)	46 (92.0)	141 (64.4)	74 (85.1)	<0.0001
Observation period (years)	2.50 (1.85–3.16)	2.41 (2.12–2.71)	2.73 (2.24–3.22)	NS
Stage of fibrosis (%)				<0.0001
F0	1 (2.0)	3 (1.4)	10 (11.5)	
F1	8 (16.0)	28 (12.8)	28 (32.2)	
F2	11 (22.0)	32 (14.6)	19 (21.8)	
F3	8 (16.0)	26 (11.9)	10 (11.5)	
F4	22 (44.0)	130 (59.3)	20 (23.0)	
Activity, n (%)				<0.0001
A0	2 (14.3)	1 (7.1)	11 (78.6)	
A1	9 (17.6)	21 (41.2)	21 (41.2)	
A2	31 (14.3)	137 (62.8)	50 (22.9)	
A3	8 (11.0)	60 (82.1)	5 (6.9)	
Blood and biochemical examination				
AST (IU/L)	39.0 ± 20.6	53.8 ± 26.6	32.0 ± 12.2	<0.0001
ALT (IU/L)	39.0 ± 21.8	51.8 ± 30.7	29.7 ± 15.2	<0.0001
ALP (IU/L)	294 ± 117	319 ± 128	306 ± 138	0.3728
Platelets ($\times 10^4/\mu L$)	14.2 ± 5.1	13.3 ± 5.7	19.0 ± 7.7	<0.0001
AFP (ng/mL)	348 ± 421	290 ± 201	915 ± 321	0.2485
DCP (mAU/mL)	542 ± 1090	805 ± 5170	2450 ± 9090	0.0770
ICG-R15 (%)	11.5 ± 8.6	17.5 ± 10.1	13.2 ± 10.8	<0.0001

Liver damage (%)				0.0016
A	46 (92)	163 (74.4)	77 (88.5)	
B	4 (8.0)	56 (25.6)	10 (11.5)	
Clinical stages (%)				0.0068
I	6 (19.3)	51 (30.7)	4 (6.9)	
II	22 (71.0)	90 (54.2)	41 (70.7)	
III	3 (9.7)	24 (14.5)	11 (19.9)	
IVa	0 (0)	1 (0.6)	2 (3.4)	
Macroscopic curability (%)				0.0096
A1	13 (26.0)	66 (30.2)	10 (11.5)	
A2	33 (66.0)	126 (57.5)	61 (70.1)	
B	4 (8.0%)	27 (12.3%)	16 (18.4%)	
Histological classification of HCC cell differentiation				1.558
Poor	8 (16.0)	40 (18.3)	16 (18.4)	
Moderate	31 (62.0)	113 (51.6)	51 (58.6)	
Well	11 (22.0)	66 (30.1)	20 (23.0)	
Histological stage				0.0044
I	13 (26.0)	62 (28.3)	7 (8.1)	
II	31 (62.0)	127 (58.0)	63 (72.4)	
III	6 (12.0)	30 (13.7)	17 (19.5)	
Tumor size (%)				0.087
<30 mm	25 (50)	129 (58.9)	21 (24.4)	

Data are presented as the mean ± SD or median with range, unless otherwise specified.
Data were analyzed using the Kruskal-Wallis and Steel-Dwass tests. HCC, hepatocellular carcinoma, HBV (+), patients with HBs-antigen (Ag) positive; HCV (+), patients with anti-HCV antibody positive; NBNC, patients without HBsAg or patients without anti-HCV antibody; AST, aspartate transaminase; ALT, alanine transaminase; ALP, alkaline phosphatase; AFP, α-fetoprotein; DCP, des-gamma carboxyprothrombin/protein induced by vitamin K absence or antagonist-II; ICG-R15, indocyanine green clearance rate at 15 min.

Table S2 Clinical profiles of patients with hepatocellular carcinoma whose frozen noncancerous liver tissues in Cohort 2 were analyzed via real-time quantitative PCR (n = 62)

Parameter	HBV (+)	HCV (+)	NBNC	P-value
N (%)	14 (22.6)	27 (43.5)	21 (33.9)	
Age (years)	59.5 (50–68)	70 (53–80)	71 (36–83)	0.0002
Sex (male, %)	12 (85.6)	18 (66.7)	18 (85.7)	0.2064
Observation time (years)	4.10 (0.31–6.04)	1.32 (0.41–4.32)	3.10 (0.25–7.05)	0.0007
Fibrosis stage, n (%)				0.0289
F0	1 (7.1)	1 (3.7)	5 (23.8)	
F1	1 (7.1)	7 (25.9)	7 (33.3)	
F2	4 (28.6)	4 (14.8)	3 (14.3)	
F3	2 (14.3)	1 (3.7)	2 (9.5)	
F4	6 (42.9)	14 (51.9)	4 (19.1)	
Activity, n (%)				0.0313
A0	3 (42.8)	2 (28.6)	2 (28.6)	
A1	4 (36.4)	3 (27.3)	4 (36.3)	
A2	9 (26.5)	13 (38.2)	12 (35.3)	
A3	0	9 (90)	1 (10)	
Blood and biochemical examinations				
AST	29.0 (16.0–60.0)	50.0 (25.0–150.0)	31.0 (18.0–80.0)	0.0068
ALT	33.0 (3.0–52.0)	43.0 (20.0–224.0)	30.0 (10.0–154.0)	0.0077
PLT	13.0 (6.9–22.1)	10.2 (4.4–44.3)	19.8 (6.2–33.3)	0.0003
AFP	7.6 (2.4–2840.0)	17.2 (1.6–23881.6)	11.4 (1.6–3173.2)	0.5851
ICGR15 (%)	9.2 (2.0–21.8)	19.0 (6.9–44.9)	11.0 (3.1–48.0)	0.0030
DCP	109.0 (12.0–4807.0)	44.0 (9.0–71246.0)	124.0 (8.0–25088.0)	0.5067
Size, <30 mm (%)	6 (42.9)	12 (44.4)	19 (90.5)	0.0009
Clinical stage, n (%)				0.2805
1	3 (21.5)	6 (22.2)	1 (4.8)	
2	9 (64.3)	10 (37.1)	11 (52.4)	
3	1 (7.1)	9 (33.3)	7 (33.3)	
4a	1 (7.1)	2 (7.4)	2 (9.5)	

Data were analyzed using the Kruskal-Wallis and Steel-Dwass tests. HBV (+), patients with HBs-antigen (Ag) positive; HCV (+), patients with anti-HCV antibody positive; NBNC, patients without HBsAg or patients without anti-HCV antibody. AST, aspartate aminotransferase; ALT, alanine aminotransferase; ALP, alanine phosphatase; γ GGT, gamma-glutamyl transpeptidase; PLT, platelet count; AFP, alpha-fetoprotein; ICGR15, indocyanine green retention rate 15 min; DCP, des-gamma-carboxy prothrombin; TP, total protein; T-Bil, total bilirubin; Alb, albumin; ChE, cholinesterase; T-Chol, total cholesterol; BUN, blood urea nitrogen; Cr, creatinine; NH_3, ammonia; HbA1c, hemoglobin A1c.

Table S3 Scoring system used for the evaluation of liver histology in this study. Formalin-fixed paraffin-embedded with Hematoxylin and eosin-stained liver sections were semi-quantitatively analyzed by assigning a score for each of the following features:

I.	*Degree of inflammatory cell infiltration*<br>*(0 for none, 1 for minimal, 2 for mild, 3 for moderate, and 4 for marked)*<br>*a) periportal; b) parenchymal; c) portal areas*
II.	*Severity of fibrosis (F stage)*<br>*(0 for F0, 1 for F1, 2 for F2, 3 for F3, 4 for F4)*
III.	*Degree of lymphoid aggregates in the portal area*<br>*(0 for none, 1 for mild, 2 for scattered, 3 for cluster, 4 for lymph follicle without germinal center, 5 for lymph follicle with germinal center)*
IV.	*Severities of portal sclerotic change, pericellular fibrosis, and steatosis*<br>*(on a scale of 0–4, with 0 for none to 4 for marked)*
V.	*Severity of damage to the bile duct*<br>*(on a scale of 0–4, with 0 for none to 4 for disappearance)*
VI.	*Severity of irregular regeneration of hepatocytes*<br>*a) anisocytosis of hepatocytes*<br>*b) bulging of hepatocytes,*<br>*c) nodular arrangement of parenchyma,*<br>*d) map-like distribution,*<br>*e) oncocytic change of hepatocytes,*<br>*f) proliferation of atypical hepatocytes*<br>*(0 for none; 1 for <1/3 of hepatocytes in the sample affected; 2 for 1/3～2/3 of the hepatocytes affected; 3 for ≧2/3 of the hepatocytes affected; 4 for all hepatocytes diffusely affected*

Table S4 Sequences of primers used for real-time reverse transcription-quantitative PCR

Genes	Sense primers (3'–5')	Antisense primers (3'-5')
CDT1	ttctccgggccagaagataaag	atgacgcaagctcagagatg
FBLN1	cctggaggccacatttgtg	tgtccacactggtagccaac
SLC30A6	tactggcttcctgcttatgtgg	acagggctaggtttcctcaatg
GPC5	gcagcagtttcttcaaacgtc	aaggccatgttcctgtaggtac
MBD3L1	acgagaattacaccccatcctg	tgaaagttctcctgcactgc
SYNPO	aggcccaactcccatctaatg	tggtggtagctggagttgatac
NLGN4X	attgacggcagcattttggc	agcccatagttgccttttgc
AGFG1	catgacaacattcacacaacagg	tttgtggatccctgaagtctgg
OR2AT4	cttttcaccacaaccactgtcc	accaggatgaaggcttctgaac
DPPA5	ttgtacaagctccggaccaag	tcatggcttcggcaagtttg
OR56A5	tatgaccgctatgtggccatc	tcctggccacaacaaagatg
OR5D13	ctgttggagaacttggttgtgg	aaacggtcataagccatcgc
ZNF98	tgtgggtattgctgcctctaag	tgctttggccaaaggtcttg
OR7D4	accaagggcaagtacaaagc	atgggtcacagcagaactcag
APOPT1	cggggaagaagacctttctcc	tcttggagggcagaatcttgag
CDY2B	tgctgcggtcttgattttgg	tccaccatttcaaggcttgc
BEND4	ttgtacctccaaccccgattg	aaaatgagccaccgttgtgc
RPL23P8	agcgttcaagatgtggaagc	tcagcacagttgatcacagc
PIGZ	tgcacccagatgagttcttcc	agctgctggggtaaaactcc
SNHG3	aggatgcttcgcgttttctc	caatgccaaaatgcgaagtgc
BCAR4	tcgactgtgattctgggactc	ttctcgtcgactgtaaccgtac
CDRT15P2	tgcctgaaatcatggtggtg	atgcctggaggagaagattgc
LOC100133612	ggcctgagagattatgtttgcg-	gtctgccccttcaaatatggc
ACTB	attcctatgtgggcgacgag	aggtgtggtgccagattttc

AGFG1, ArfGAP with FG repeats 1; APOPT1, cytochrome c oxidase assembly factor 8 (COA8); ACTB, β-actin: BCAR4, breast cancer anti-estrogen resistance 4; BEND4, BEN domain containing 4; CDRT15P2, CMT1A duplicated region transcript 15 pseudogene 2; CDT1, chromatin licensing and DNA replication factor 1; CDY2B, chromodomain Y-linked 2B; DPPA5, developmental pluripotency associated 5; FBLN1, fibulin 1; SLC30A6, solute carrier family 30 member 6; GPC5, glypican 5; LOC100133612: LOC1134 (Gene ID: 100133612), long intergenic nonprotein coding RNA 1134; NLGN4X, neuroligin 4 X-linked; MBD3L1, methyl-CpG binding domain protein 3 like 1; OR2AT4, olfactory receptor family 2 subfamily AT member 4; OR56A5, olfactory receptor family 56 subfamily A member 5; OR5D13, olfactory receptor family 5 subfamily D member 13; OR7D4, olfactory receptor family 7 subfamily D member 4; PIGZ, phosphatidylinositol glycan anchor biosynthesis class Z; RPL23P8, ribosomal protein L23 pseudogene 8; SNHG3, small nucleolar RNA host gene 3; SYNPO, synaptopodin; ZNF98, zinc finger protein 98.

Supplementary Figures

Supplementary Figures S1

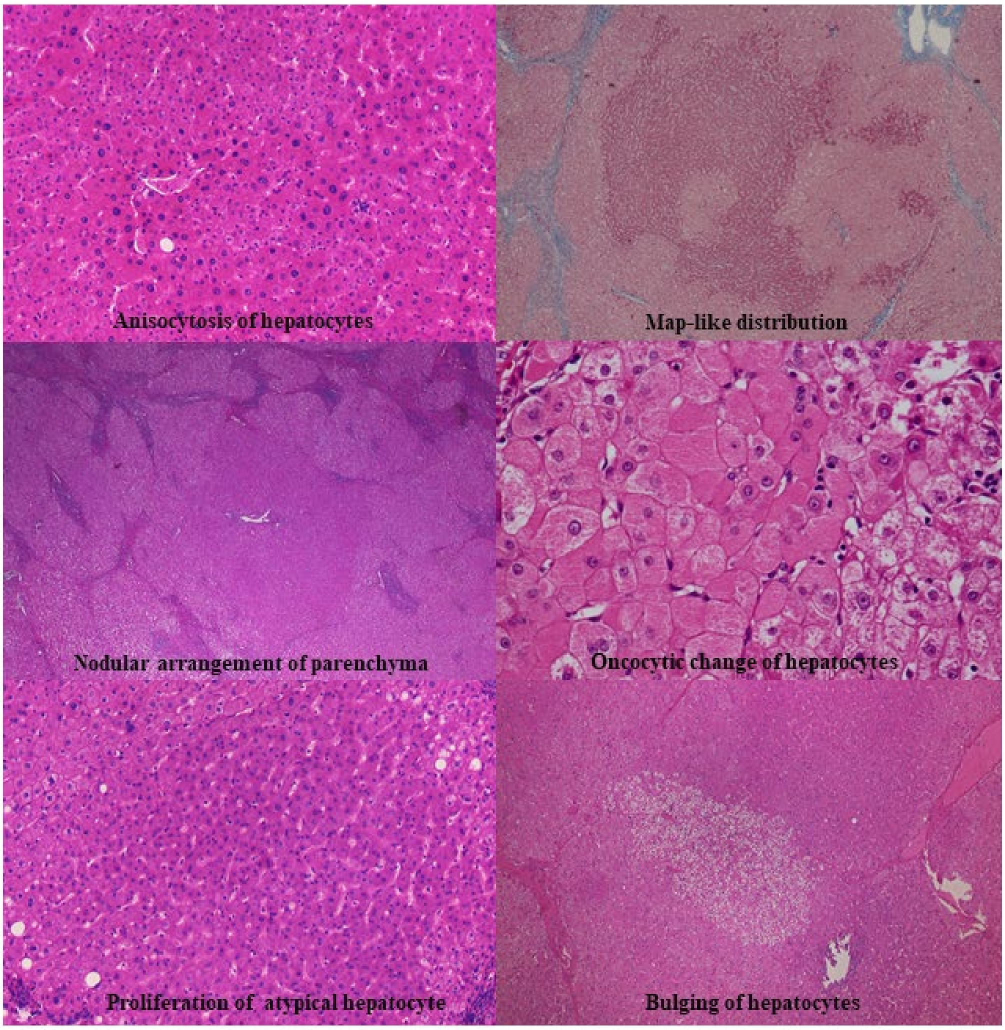

Supplementary Figure S1

Representative hematoxylin and eosin (HE) staining images of the irregular regeneration (IR) of hepatocyte in noncancerous liver sections. Repeated necro-inflammatory reactions in the lobules cause irregular hepatocyte necrosis and regeneration over time. As a result, irregular regenerative hepatocytes with various morphological changes intermingle with each

hepatocyte region in the lobules. Thus, the irregular regeneration (IR) of hepatocytes represents a state in which hepatocytes lose their normal homogeneity and uniformity. The IR of hepatocytes consisted of anisocytosis of hepatocytes characterized by variable cell size with focal dysplastic changes (HE) staining, ×100 magnification); map-like distribution of a distinct population of hepatocytes with a homogenous appearance within each population, separated from each other by a sharp outline (HE staining, ×40 magnification); nodular arrangement of parenchyma (HE staining, ×40 magnification); oncocytic change of hepatocytes characterized by extreme pleiomorphism (HE staining, ×400 magnification); proliferation of atypical hepatocytes characterized by hepatocytes with a high nuclei/vesicle ratio and a large amount of chromatin aggregated in the parenchyma (HE staining, ×100 magnification); bulging of hepatocytes characterized by extensive proliferation of hepatocytes compressing the surrounding parenchyma (HE staining, ×40 magnification).

Supplementary Figures S2

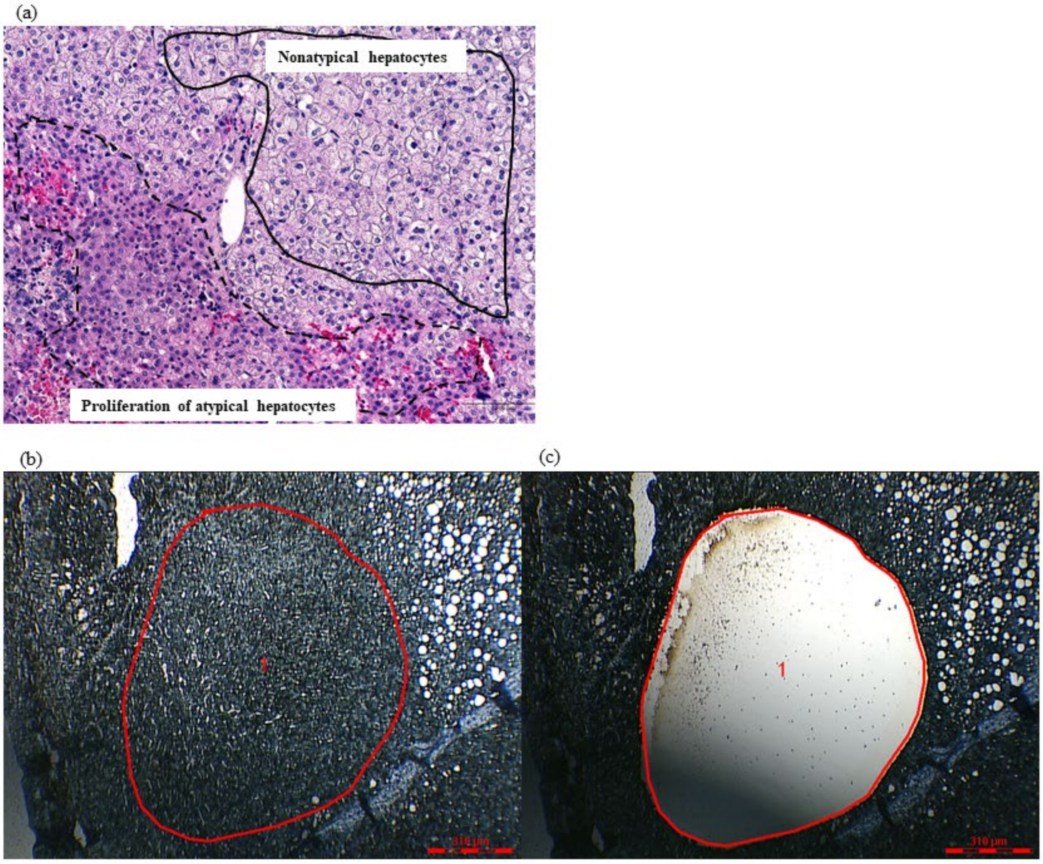

Supplementary Figure S2 Laser-capture microdissection (LCM) was performed using LMD6500 system (Leica Microsystems, Wetzlar, Germany). (a) Cells excised using LCM are shown. The resected area along the straight line contains atypical hepatocytes, and the resected area along the dashed line contains nonatypical hepatocytes. Hematoxylin and eosin (HE) staining, ×100. LCM images taken (b) before and **(c)** after Toluidine blue staining of formalin-fixed paraffin-embedded tissues. Both aggregations of candidate hepatocytes and areas without candidate hepatocytes in the same tissue were chosen as target cells (left image), and dissection was performed immediately (right image). The dissected tissues were collected using LCM on an LMD6500 system (Leica Microsystems).

Supplementary Figures S3

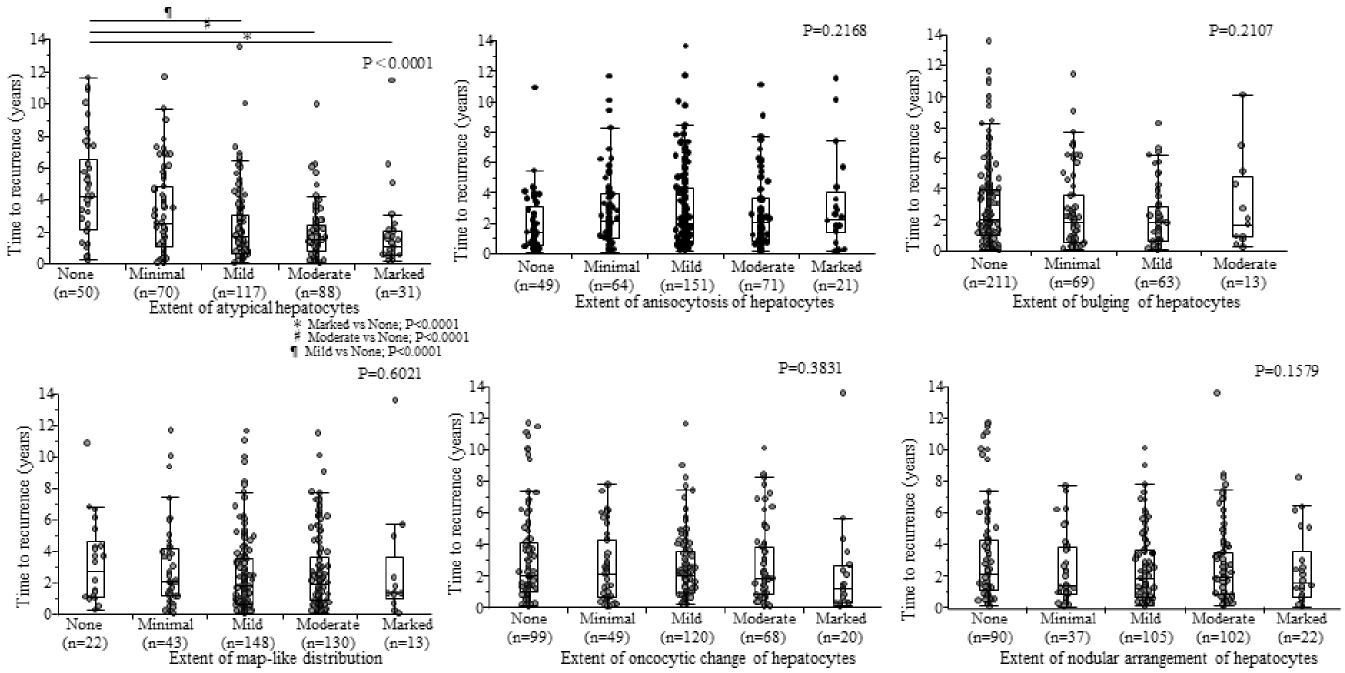

Supplementary Figures S3

Association between time to postoperative recurrence and the extent of atypical hepatocytes in noncancerous hematoxylin and eosin (HE)-staining sections in Cohort 1 (none versus (vs.) mild, $p < 0.0001$; none vs. moderate, $p < 0.0001$; none vs. marked, $p < 0.0001$). Anisocytosis of hepatocytes ($p = 0.2168$); bulging of hepatocytes ($p = 0.2107$); map-like distribution ($p = 0.6021$); oncocytic change of hepatocytes ($p = 0.3831$); nodular arrangement of hepatocytes ($p = 0.1579$). The Kruskal-Wallis and Steel-Dwass tests were performed for comparisons among multiple groups.

Supplementary Figures S4

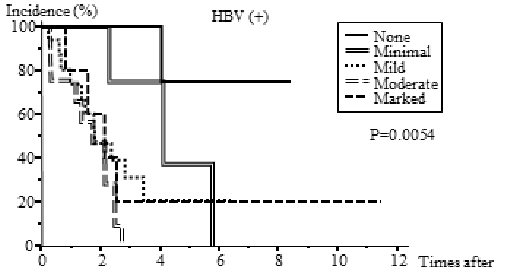

None	9	9	8	6	5	3	3	3	2			
Minimal	7	7	5	4	3	2						
Mild	16	12	7	5	3	2	2					
Moderate	13	9	6	1								
Marked	5	5	4	2	1	1	1	1	1	1	1	1

scores	n	Event	5-year survival	Hazard ratio (95% CI)	P
0	9	1	75.0%	1.00	
1	7	3	0%	4.93 (0.63-99.8)	0.130
2	16	11	20.8%	10.38 (2.00-190.3)	0.0026
3	13	11	0%	22.7 (4.19-423.9)	<0.0001
4	5	4	20.0%	10.20 (1.50-200.0)	0.0164

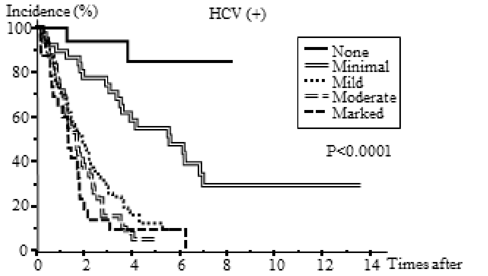

None	21	18	16	13	10	5	3	3	2					
Minimal	42	34	26	25	18	17	14	7	5	5	3	3	2	1
Mild	85	58	37	21	10	5								
Moderate	48	33	16	7	4									
Marked	23	15	6	4	3	3	2							

scores	n	Event	5-year survival	Hazard ratio (95% CI)	P
0	21	2	84.38%	1.00	
1	42	21	55.0%	4.66 (1.36-29.1)	0.010
2	85	66	9.0%	15.4 (4.79-94.15)	<0.0001
3	48	40	0%	19.9 (6.06-123.0)	<0.0001
4	23	21	9.1%	23.2 (6.77-146.0)	<0.0001

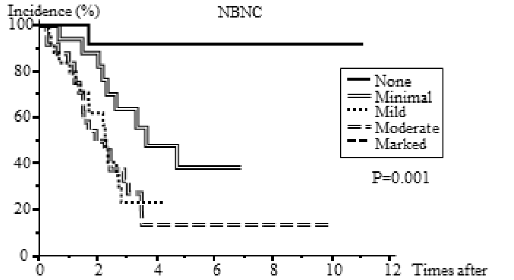

None	13	13	12	12	10	9	6	5	3	3	2	1
Minimal	21	17	16	9	7	4	3					
Mild	26	21	13	4	2							
Moderate	25	22	13	8	3	2	2	2	2	2	2	
Marked	2	1										

scores	n	Event	5-year survival	Hazard ratio (95% CI)	P
0	13	1	91.67%	1.00	
1	21	9	38.24%	0.05 (1.69-167.2)	0.006
2	26	16	0%	- -	-
3	25	19	13.7%	21.6 (4.23-395.5)	<0.0001
4	2	0	0%	- -	-

Supplementary Figure S4 Cumulative relapse-free survival rates after the first liver resection for HCC in Cohort 1. Patients with hepatitis B virus (HBV) surface antigen (HBsAg) [HBV(+)], Patients with anti-hepatitis C virus (HCV) [HCV(+)], and patients without HBsAg or anti-HCV [NBNC]. Data were analyzed using the Kaplan–Meier method, and differences among groups were analyzed using the log-rank test.

Supplementary Figure S5 3D file

Please view to power point file

Supplementary Figure S5 360˚ view movies of 3D reconstructed images by confocal scanning microscopy. (A) Colocalization of CDT1 and Ki-67 were observed in a nuclear in hepatocytes. (B) Colocalization of CDT1 and Ki-67 were observed in a nuclear in cancer cell. Blue, nuclei counterstained with Hechest33342; blue, CDT1; green, Ki-67; red and Merged image; Orange.

Supplementary Figure S6

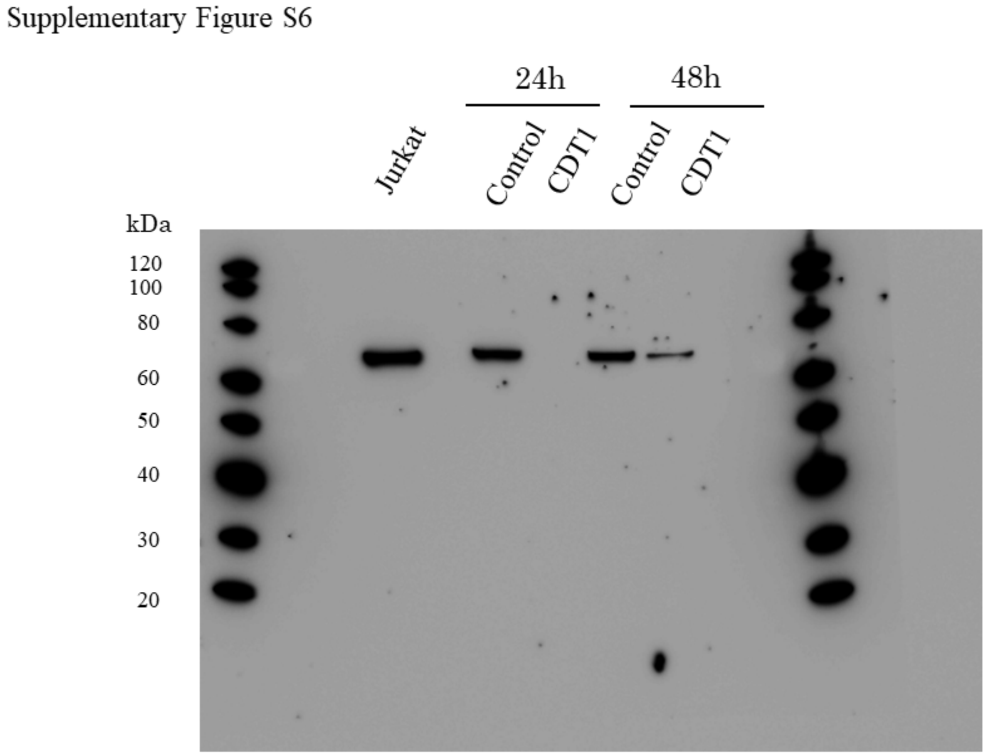

The figure is a photograph of CDT1s protein expression levels compared to Figure 9a. This is a photograph with lower exposure conditions compared to Figure 9a for a same gel.

Supplementary Figure S7

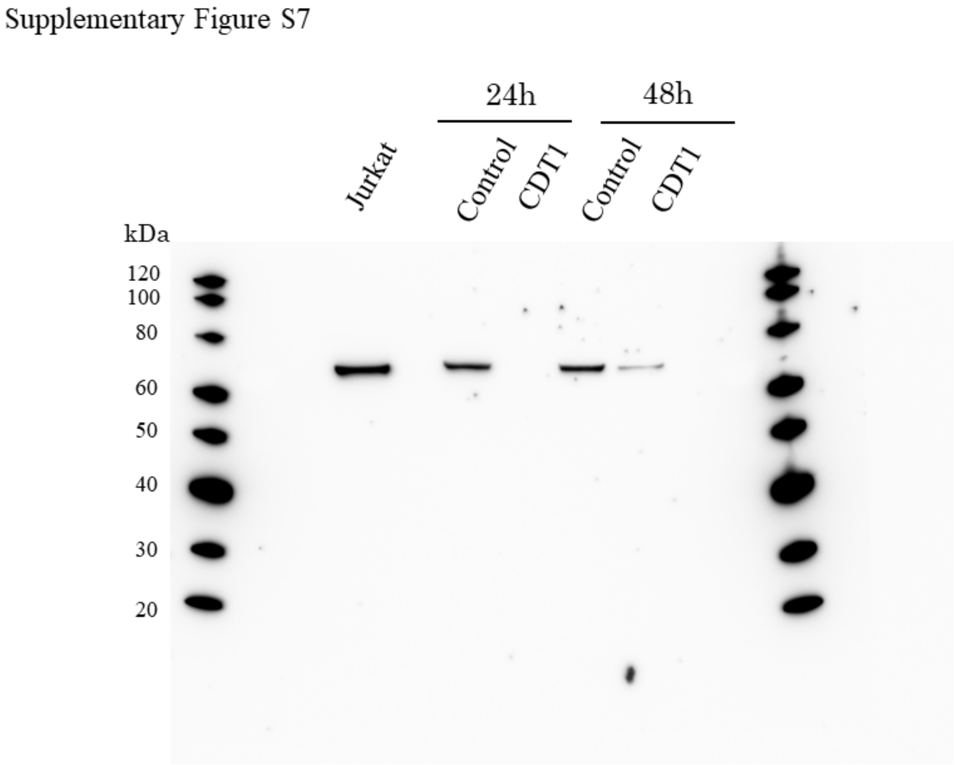

The figure is a photograph of CDT1s protein expression levels compared to Figure 9a. This is a photograph with higher exposure conditions compared to Figure 9a for a same gel.

Supplementary Figure S8

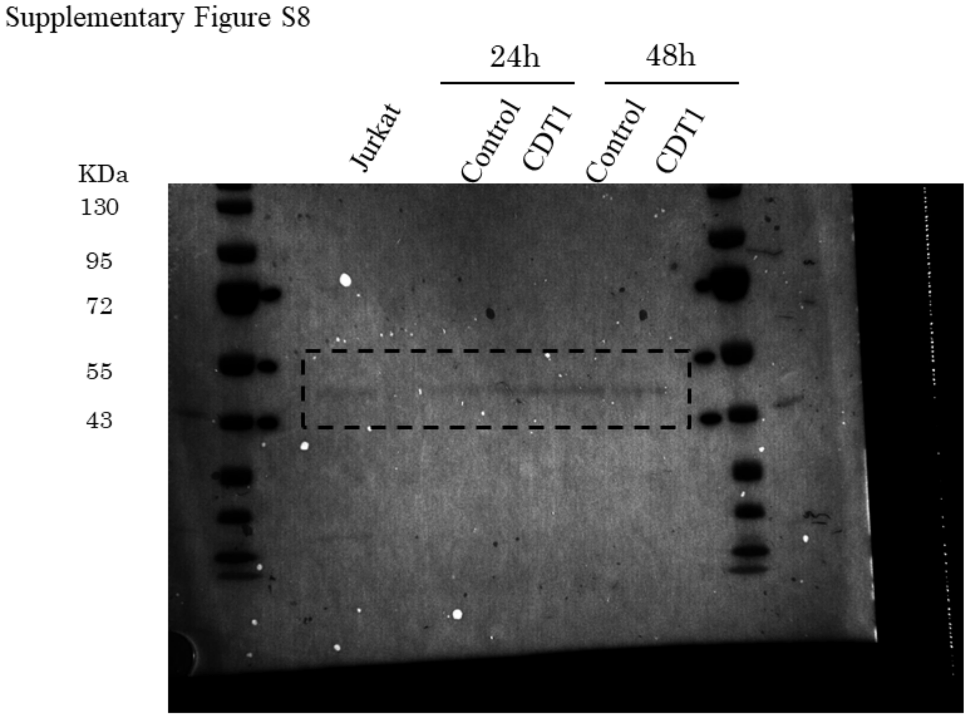

The figure is a photograph of CDT1s protein expression levels compared to Figure 9a. This is a photograph with lower exposure conditions compared to Figure 9b for a same gel.

Supplementary Figure S9

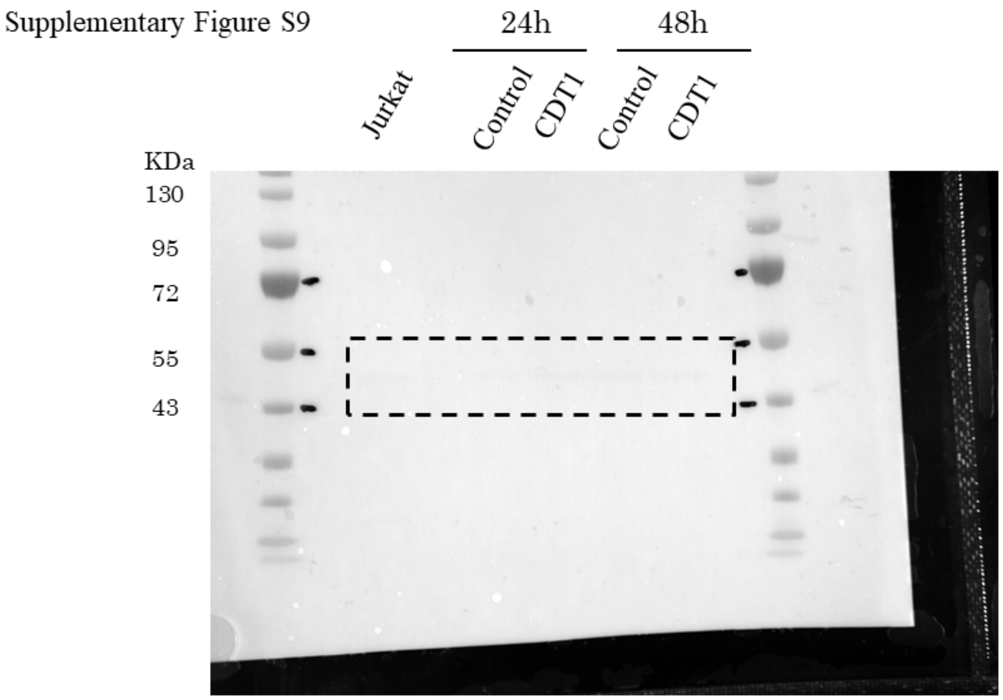

The figure is a photograph of CDT1s protein expression levels compared to Figure 9a. This is a photograph with higher exposure conditions compared to Figure 9b for a same gel.
