# Supplementary figures and images for: The proliferation of atypical hepatocytes and CDT1 expression in noncancerous tissue are associated with the postoperative recurrence of hepatocellular carcinoma
Source: Sci Rep. 2022 Nov 28;12:20508. doi: 10.1038/s41598-022-25201-6 (PMC9705552; doi:10.1038/s41598-022-25201-6)

## Slide 1
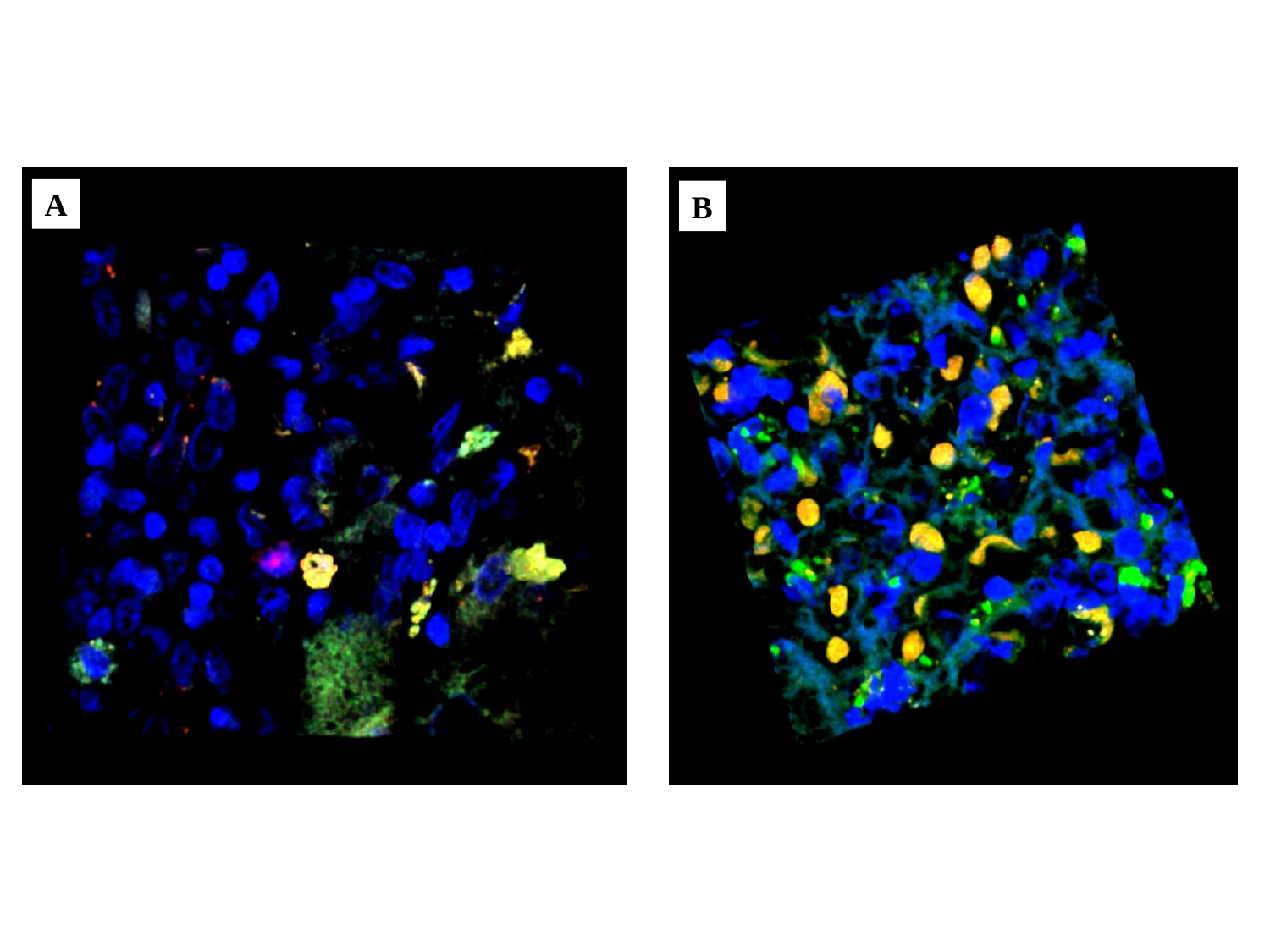

A
B

Supplement: Supplementary file 2 — Supplementary Information 2. [file 41598_2022_25201_MOESM2_ESM.pptx]
